# Design, synthesis, and biological assessment of a novel series of coumarin-tethered thiazole derivatives as potential antibacterial agents

**DOI:** 10.3389/fchem.2025.1627186

**Published:** 2025-09-05

**Authors:** Manal S. Ebaid, Hanaa Farag, Mohamed Abdelraof, Abdulrahman M. Saleh, Mohamed G. Thabit, Jarosław Dziadek, Ahmed A. Youssef, Ahmed Sabt

**Affiliations:** 1 Department of Chemistry, College of Science, Northern Border University, Arar, Saudi Arabia; 2 Pesticide Chemistry Department, Chemical Industries Research Institute, National Research Centre, Cairo, Egypt; 3 Microbial Chemistry Department, National Research Centre, Biotechnology Research Institute, Giza, Egypt; 4 Department of Pharmaceutical Chemistry, Faculty of Pharmacy, Cairo University, Cairo, Egypt; 5 Pharmaceutical Chemistry Department, Faculty of Pharmacy, Pharos University (PUA), Alexandria, Egypt; 6 Laboratory of Genetics and Physiology of Mycobacterium, Institute of Medical Biology of the Polish Academy of Sciences, Lodz, Poland; 7 Department of Biochemistry, Faculty of Pharmacy, Egyptian Russian University, Cairo, Egypt; 8 Department of Laboratory Science, College of Pharmacy, University of Kut, Wasit, Iraq; 9 Chemistry of Natural Compounds Department, Pharmaceutical and Drug Industries Research Institute, National Research Centre, Cairo, Egypt

**Keywords:** Coumarin-thiazole derivatives, Antibacterial activity, Drug Resistance, Biofilm, DNA Gyrase, molecular docking, molecular dynamics

## Abstract

Since the discovery of penicillin in the 1930s, antibiotics have been the primary treatment for bacterial infections. However, antimicrobial resistance (AMR) has escalated due to antibiotics overuse and misuse. To address this concern, a new series of coumarin-thiazole derivatives was synthesized and evaluated against *Serratia fonticola, Campylobacter jejuni, Enterococcus faecalis, and Achromobacter xylosoxidans*. Most compounds showed selective activity, with compounds 6a and 6c exhibiting potent effects against *E. faecalis* (MICs: 25, 12.5 μg/mL) and *A. xylosoxidans* (MICs: 50, 25 μg/mL), comparable to ciprofloxacin. Further studies revealed that 6a and 6c effectively disrupted bacterial biofilms with a low resistance risk. Mechanistically, they induced ROS production, thereby impairing redox homeostasis and reducing lipid peroxidation. Additionally, compound 6a inhibited *E. coli* DNA gyrase (IC_50_ = 23.75 μg/mL). Molecular docking studies (PDB ID: 4duh) and dynamics simulations confirmed the stable binding of these compounds to DNA gyrase, suggesting their potential as novel antibacterial agents. These findings highlight promising avenues for the development of new therapeutic agents to combat AMR.

## 1 Introduction

Infectious diseases remain a leading cause of global mortality, with antimicrobial resistance (AMR) posing a critical threat to public health (Naghavi et al., 2024). The rise of multidrug-resistant (MDR) bacterial strains has rendered many conventional antibiotics ineffective, leading to increased morbidity and mortality (Llor and Bjerrum, 2014; Prestinaci et al., 2015). In 2019 alone, AMR was associated with nearly 5 million deaths, underscoring the urgent need for novel therapeutic strategies (Antimicrobial Resistance Collaborators, 2022; Salam et al., 2023). Despite ongoing research efforts, the development of new antibiotics has been hindered by toxicity, limited efficacy, and rapid resistance emergence (Huemer et al., 2020; Ayukekbong et al., 2017; Prestinaci et al., 2015). As a pharmaceutical chemist, I propose a multifaceted approach to combat AMR through innovative drug design, alternative antimicrobial agents, and strategic resistance mitigation (Lungu et al., 2023; Silver, 2011). The development of drug resistance remains a critical challenge in pharmaceutical chemistry, particularly in antimicrobial and anticancer therapies. Single-target agents are highly susceptible to resistance due to the ease with which pathogens or cancer cells can mutate or activate alternative pathways (Sabt et al., 2023; Sabt et al., 2024a; Sabt et al., 2024b). In contrast, multi-targeting agents—particularly those designed via molecular hybridization—demonstrate superior efficacy and a reduced likelihood of resistance development (Singh H. et al., 2017).

**FIGURE 1:**
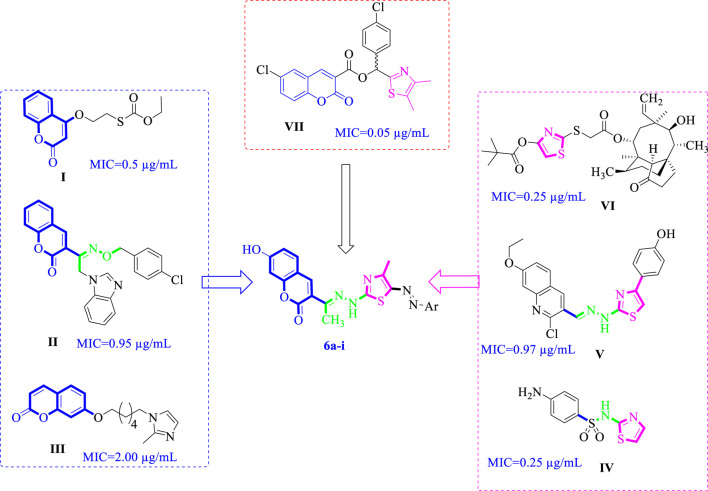
Reported antibacterial agents containing bioactive cores coumarin I-III, thiazole IV-VI, Coumarin-thiazole VII and our newly target compounds 6a-i.

Natural products have been utilized for the treatment of diseases for millennia and are becoming increasingly significant in the realms of drug discovery and development. Notably, a substantial proportion of anti-infective agents are derived from natural sources (Li et al., 2013; Weinmann, 1997). Coumarin, a natural product and the simplest representative of the oxygen-containing heterocycles known as benzo [*b*]pyran-2-one, is a secondary metabolite found in various plant families, including *Apiaceae*, *Fabaceae*, *Asteraceae*, *Solanaceae Rubiaceae*, *Rosaceae*, and *Rutaceae* (Sharifi-Rad et al., 2021). Compounds containing a coumarin nucleus have been documented to demonstrate a variety of biological functions, such as anticoagulant, anti-fungal, anticancer, antileishmanial, antiviral effects in addition to carbonic anhydrase inhibitors (Batran et al., 2023; Hassan et al., 2023; Ibrahim et al., 2022; Koley et al., 2024; Sabt et al., 2024c). Furthermore, researchers have focused on the antimicrobial properties of coumarins (Bhagat et al., 2019; Cheke et al., 2022; Hu et al., 2018; Ranjan et al., 2021). For instance, Coumarin−carbonodithioate hybrid I exhibited significant antibacterial efficacy, with MICs of 0.5 μg/mL against *S. aureus* and *B. subtilis*, and 1 μg/mL against *E. coli* and *P. aeruginosa* (Mangasuli et al., 2018). Similarly, the coumarin-benzimidazole hybrid II demonstrated significant antibacterial agent with a wide range of efficacy against *Staphylococcus aureus, Pseudomonas aeruginosa, Proteus vulgaris and Bacillus subtilis*. Importantly, this compound exhibited no cytotoxic effects or hemolytic activity at concentrations 10-fold greater than the MIC (Singh L. R. et al., 2017). Additionally, the coumarin-imidazole derivative III revealed strong and extensive antimicrobial activity (Hu et al., 2018) (Figure 1).

It has been reported that the thiazole moiety serves as a crucial element in numerous natural compounds, including vitamin B (thiamine) and antibiotics such as penicillin (Eryılmaz et al., 2020). Moreover, the thiazole nucleus has demonstrated a diverse array of pharmacological activities, including antileishmanial (Queiroz et al., 2020), antiviral (Farghaly et al., 2022), anticancer (Sabt et al., 2018), antidiabetic (Abid et al., 2025), and significant antibacterial properties (Ahmad et al., 2020; Sayed et al., 2023) (e.g., compound IV) (Figure 1) (Qi et al., 2025). Additionally, thiazole nucleus can possess the capability to inhibit several enzymes, such as carbonic anhydrase (Karakaya et al., 2024) and dihydrofolate reductase, as inhibited by compound V (Figure 1) (Ammar et al., 2021). Previous studies have indicated that thiazole can impede bacterial growth by disrupting the biosynthesis of specific bacterial lipids, as exemplified by sulphathiazole VI (Figure 1) (Desai et al., 2013).

It is worth noting that thiazoles and similar N-heterocycles (e.g., triazoles, oxazoles) are widely used in organic synthesis and medicinal chemistry (Joule and Mills, 2010; Klika et al., 2021; Klika et al., 2022). DNA gyrase is classified within the DNA topoisomerase protein family and is responsible for facilitating the interconversion of various topological forms of DNA. This enzyme is crucial for processes such as DNA replication and transcription, as well as other cellular functions (Rajakumari et al., 2024). Consequently, the inhibition of DNA gyrase presents a promising target for the development of antibacterial agents. For instance, compounds such as coumarin-thiazole VII specifically inhibit DNA gyrase, highlighting the enzyme’s importance in combating bacterial infections (Liu et al., 2020).

This study focuses on the design and synthesis of new coumarin-based derivatives conjugated with thiazole scaffolds, aiming to incorporate diverse pharmacophoric features into a single molecular framework. The primary goal of this approach was to develop innovative coumarin-thiazole hybrids with enhanced antibacterial activity. The synthesized compounds were evaluated against multiple bacterial strains, with minimum inhibitory concentration (MIC) assays and antibacterial efficacy assessments conducted for the most promising candidates. Furthermore, their potential to disrupt bacterial biofilms was investigated. To explore the mechanistic basis of their antibacterial action, preliminary studies were performed, focusing on intracellular oxidative stress markers, including reactive oxygen species (ROS) production and lipid peroxidase activity. The most active derivatives were further analyzed for their inhibitory effects on the DNA gyrase enzyme. To elucidate binding interactions and target engagement, molecular docking simulations were employed, supported by molecular dynamics studies to validate the docking results and provide deeper insights into the binding stability.

## 2 Results and discussion

### 2.1 Chemistry

**SCHEME 1:**
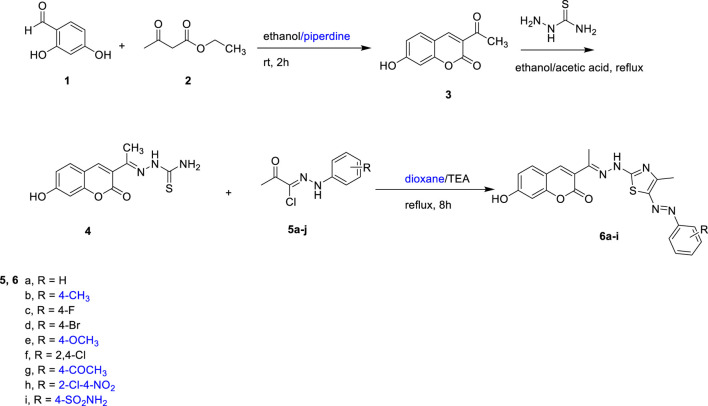
Synthesis of the target compounds 6a-i.

The synthesis of coumarin derivatives 6a-i was executed in accordance with the methodologies delineated in Scheme 1, which also illustrates the formation of the precursor compound, 3-acetyl-7-hydroxy-2*H*-chromen-2-one (3). In this investigation, a Knoevenagel condensation reaction was employed, wherein 2,4-dihydroxybenzaldehyde (1) was reacted with ethyl acetoacetate (2) at ambient temperature, facilitated by a minimal quantity of piperidine in ethanol, yielding 3-acetyl-7-hydroxy-2*H*-chromen-2-one (3) (Hassan et al., 2023). Subsequently, this compound underwent reflux with thiosemicarbazide in absolute ethanol, with the addition of several drops of glacial acetic acid, resulting in the formation of the corresponding thiosemicarbazone 4. The heterocyclization of thiosemicarbazone derivative 4, an important intermediate, was successfully accomplished by refluxing it with a range of hydrazonoyl chloride derivatives 5a-I. These derivatives were synthesized using established methods that involve the reaction of diazonium chloride with appropriately activated halogenated methylene groups, which is an efficient approach for generating various hydrazonoyl halides (Osman et al., 2023). The reaction was conducted in dioxane, with the addition of a few drops of triethylamine (TEA), resulting in the formation of the desired compounds, 6a-i. The structural characterization of the newly synthesized derivatives was performed utilizing ^1^H-NMR and ^13^C-NMR spectroscopy, as detailed in the Experimental section.

### 2.2 Biological activity

#### 2.2.1 Antibacterial screening

Recently, the drug-resistant bacteria represented a distinctive hindrance, especially those having the capability to proliferate in the presence of several antibiotic groups (Matin et al., 2019). The resistance of bacterial pathogens toward at least one agent in three or more antibacterial families was characterized as MDR. Moreover, the bacterial pathogen was called XDR when it’s non-susceptible to at least one agent in all but two or fewer antimicrobial categories. In this regard, our vision is aimed at investigating the ability of the targeted compounds to cause bactericidal effects against the clinically important bacterial pathogens. In which, these models were isolated from different urinary tract infected samples, which had a significant resistance toward the classical antibacterial agents as mentioned in the antibiotic profile for each pathogen (Table 1). The new coumarin-based derivatives conjugated with the thiazole scaffold were examined as a potential drug against these pathogens due to the potential antibacterial activity of coumarin and thiazole derivatives.

**TABLE 1:** Antibacterial activity of the targeted molecules using agar-well diffusion.

Compound	Inhibition zone (mm)			
	*Serratia fonticola* (Gram-negative)	*Campylobacter jejuni* (Gram-positive)	*Enterococcus faecalis* (Gram-positive)	*Achromobacter xylosoxidans* (Gram-negative)
6a	2 ± 0.02	ND	6 ± 1.02	4 ± 1.11
6b	ND	ND	5 ± 0.38	5 ± 0.82
6c	2 ± 0.16	ND	7 ± 0.44	5 ± 0.25
6d	ND	ND	5 ± 0.18	4 ± 0.31
6e	ND	ND	5 ± 0.52	3 ± 0.73
6f	ND	ND	4 ± 0.61	2 ± 0.12
6g	ND	ND	ND	ND
6h	2 ± 0.31	ND	2 ± 0.07	3 ± 0.91
6i	ND	ND	2 ± 0.21	ND
Ciprofloxacin^a^	4 ± 0.56	3 ± 0.42	4 ± 0.78	5 ± 0.22
Cephradine	ND^b^	ND	ND	ND
Ampicillin	ND	ND	ND	2 ± 0.01
Polymexine B	3 ± 0.12	ND	3 ± 0.29	4 ± 0.65
Kanamycin	ND	2 ± 0.06	ND	ND
Erythromycin	ND	ND	3 ± 0.15	2 ± 0.18

^a^ Kanamycin, Ciprofloxacin, Ampicillin, Polymexine B, kanamycin, Erythromycin and Cephradine were used as standard antibacterial agents at 20 μg/mL.

^b^ ND: not determined.

The target compounds 6a-i were prepared, and their antibacterial activities were investigated against XDR and MDR pathogens. As can be seen in Supplementary Figure S1, the sensitivity of the XDR pathogens (*C. jejuni* and *S. fonticola*) towards the tested compounds was found to be negligible, as no inhibition zone appeared around all tested molecules. High resistance demonstrated by these pathogens emphasized the ineffective action of the tested compounds; hence, those were excluded from our examination. Interestingly, the efficiency of the targeted compounds was notably performed toward the MDR pathogens (*A. xylosoxidans*) and XDR (*E. faecalis*) (Table 1). A significant activity was clearly indicated in the case of most tested compounds against both pathogens except for compound 6g. On the other hand, the activity of some tested compounds was relatively preferred when compared to the standard antibacterial agents. Since some compounds of this group obtained an inhibition zone higher than that carried out by the potent antibacterial standards. Surprisingly, compounds 6a-e had higher sensitivity towards *E. faecalis* than that obtained by ciprofloxacin (4 mm). In addition, the ability of these compounds to inhibit *A. xylosoxidans* was observed in the equal efficacy of the reference drug (5 mm) as occurred in compounds 6b and 6c. Our results clearly showed that compounds 6a-f and h were very effective against *A. xylosoxidans* and *E. faecalis*. Therefore, further studies using potent compounds were implemented against *A. xylosoxidans* and *E. faecalis* to establish more details about their activity.

The ability of the potent compounds to inhibit the targeted bacterial pathogens at the lowest concentration (MIC) could be determining the optimal dosage that maximizes the killing of bacterial colony-forming units (CFUs). Accordingly, stock solutions of each potent compound were prepared at different concentrations and incubated with each bacterial pathogen. Bacterial growth was measured using turbidimetric methods, and the percentage of survival inhibition was calculated based on the reduction of CFUs. The activity of the potent compounds 6a and 6b was clearly observed at significant MIC values, which were sometimes close to the values of the standard antibiotic Ciprofloxacin (see Table 2). Comparatively, the potency of the most active compounds was significantly greater against *E*. *faecalis* than against *A*. *xylosoxidans*, indicating the specificity of these compounds in combating *E*. *faecalis*. Notably, compound 6c demonstrated the highest MIC values, with 12.5 μg/mL against *E. faecalis* and 25 μg/mL against *A. xylosoxidans*. Overall, the superiority of compounds 6a and 6c, with MIC values not exceeding 50 μg/mL, positions them as promising candidates for application in the pharmaceutical industry.

**TABLE 2:** Minimum inhibitory concentration (MIC) of the potent synthesized derivatives.

Sample no.	Minimum inhibitory concentration (MIC, µg/mL)	
	*Enterococcus faecalis* (Gram-positive)	*Achromobacter xylosoxidans* (Gram-negative)
6a	22.8 ± 2.66	46.9 ± 3.82
6b	49.1 ± 0.79	162.3 ± 2.99
6c	10.9 ± 2.16	24.6 ± 0.36
6d	48.7 ± 2.11	47.9 ± 2.87
6e	49.2 ± 0.77	128.4 ± 3.41
8f	47.8 ± 2.58	173.4 ± 1.11
Ciprofloxacin	22.8 ± 0.25	18.5 ± 1.56

#### 2.2.2 Biofilm activity assay

Bacterial biofilms are slimy structures created by the aggregation of bacteria that can protect bacterial cells from outside influences and enhance resistance to antimicrobial agents (Yang et al., 2022). Commonly, microbial pathogens utilize this mechanism to increase their virulence and evade adverse conditions. The assessment of biofilm formation by the treated bacterial pathogens was performed using both qualitative and quantitative methodologies.

Preliminarily, the evaluation of bacterial biofilm formation was conducted at a fixed concentration of 50 μg/mL (which considered an effective concentration for the potent molecules) for all potent molecules using a Congo red plate assay. After incubating each bacterial pathogen with the targeted molecule, the resulting bacterial suspension was cultivated on the Congo red plate and incubated for 48 h. The colonial growth was then observed. Notably, the black pigment of the growth colonies was clearly visible in the untreated samples after 24 h Supplementary Figure S2. Variable antibacterial activity was observed in all treated samples, indicating the efficacy of the tested compounds at this concentration, particularly compounds 6a and 6c. The bacterial cells appeared stressed and exhibited difficulty in growth. Furthermore, the black pigment did not develop in any of the treated samples, with only a slight appearance noted in the cases of P25 and P100.

Indeed, the qualitative assay demonstrated that compounds 6c and 6a have the potential to act as strong antagonists against bacterial biofilm formation, particularly against *E. faecalis* (Supplementary Figure S2).

Quantitative assay methodologies were utilized to assess the effectiveness of the potent compounds 6a and 6c in inhibiting biofilm formation by specific bacterial pathogens. Initially, the assessment of microbial biofilm formation was conducted at a fixed concentration of 50 μg/mL. The target compound 6c demonstrated greater effectiveness against both bacterial pathogens, as shown in Table 3. Both compounds yielded significant results regarding the inability of bacterial pathogens to produce biofilm while maintaining cell proliferation without negative consequences. The assessment of biofilm formation demonstrated a significantly enhanced ability to suppress biofilm formation in both Gram-positive and Gram-negative bacteria compared to the standard drug. The percentage of bacterial biofilm inhibition exceeded 50% for *E. faecalis*, indicating that it is a promising antibiofilm agent. Conversely, it is noteworthy that 6a exhibited moderate antibiofilm efficacy against *A. xylosoxidans*, with inhibition levels below 40%. The observed differences in biofilm production among the tested pathogens in the presence of these potent molecules may be attributed to variations in the composition of the embedded biofilm.

**TABLE 3:** Antibiofilm activity of the potent molecules at MIC value.

Sample no.	Biofilm inhibition (%)	
	*Enterococcus faecalis* (Gram-positive)	*Achromobacter xylosoxidans* (Gram-negative)
6a	46.57 ± 2.06	30.21 ± 0.96
6c	55.03 ± 3.11	41.81 ± 2.85
Ciprofloxacin	66.21 ± 0.56	48.33 ± 0.56

#### 2.2.3 Intracellular oxidative stress

##### 2.2.3.1 The generation of reactive oxygen species (ROS)

The generation of intracellular oxidative stress, characterized by the presence of reactive oxygen species (ROS), has been identified as a significant factor influencing the antibacterial effectiveness of various antibiotics (Sui et al., 2021). To determine whether compounds 6a and 6c cause membrane damage that results in oxidative stress, the levels of intracellular ROS were measured using a fluorometric assay employing DCFH-DA dye. The findings revealed that the treatment of *Enterococcus faecalis* and Achromobacter xylosoxidans with compounds 6a and 6c resulted in a notable increase in intracellular ROS production, as demonstrated in Table 4. The results displayed that the highest levels of DCF detected in *E*. *faecalis* demonstrated a substantial induction of ROS by compound 6c, followed closely by compound 6a. Importantly, the ROS release was found to exceed that induced by ciprofloxacin, indicating a pronounced oxidative stress response triggered by these compounds in *E. faecalis*. Conversely, the intracellular ROS levels in *A xylosoxidan*s were significantly lower in comparison to the reference drug. Additionally, an increase in ROS was particularly noted in the context of Gram-positive bacteria.

**TABLE 4:** ROS determination of the potent molecules inside the bacterial cell’s pathogens.

Sample No	ROS determination (DCF level, counts) using spectrofluorometric (excitation and emission wavelength at 485 nm and 530 nm, respectively)	
	*Enterococcus faecalis* (Gram-positive)	*Achromobacter xylosoxidans* (Gram-negative)
6a	268.79 ± 1.76	178.02 ± 0.88
6c	284.54 ± 3.92	136.19 ± 2.17
Ciprofloxacin	277.93 ± 1.52	217.57 ± 2.16
Control	89.53 ± 0.99	97.36 ± 2.02

##### 2.2.3.2 Lipid peroxidation (LPO)

Malondialdehyde (MDA) is a crucial parameter that reflects the antioxidant potential of organisms and the extent of cellular peroxidation damage (Cui et al., 2016). The cell membrane is one of the most vital components of bacterial structure due to its role in protecting against external inhibitors. Disintegration of the cell membrane inevitably leads to the death of bacterial cells, as it facilitates the inhibition of protein and nucleic acid synthesis. The oxidation of fatty acid content is considered a significant marker indicating the disruption of the cell membrane. Therefore, investigating the lipid oxidation levels in each bacterial pathogen by comparing them with untreated samples can provide valuable insights into the pathways through which targeted molecules are eradicated.

As shown in Table 5, The oxidation potential associated with the bacterial cell membrane by the potent molecules 6a and 6c dramatically increased against both pathogens, particularly in the case of *E. faecalis*. A notable difference in LPO activity was observed among the most active compounds; the lipid oxidation level in Gram-positive *E. faecalis* was remarkably close to that observed with the standard antibiotic. However, the effect of the targeted compounds on the fatty acid content of Gram-negative *A. xylosoxidans* was found to be lower than that caused by the standard antibiotic, except for compound 6a, suggesting their efficacy against Gram-positive bacteria. Furthermore, the cell membrane structure of each bacterial type reflects the performance of the potent compounds. Notably, the ability of both compounds to induce significant oxidation of lipid content in *E. faecalis* was greater than in *A. xylosoxidans*, attributed to differences in cell membrane composition. The variations in cell membrane composition were discussed, which may be due to the presence of a lipopolysaccharide layer surrounding the Gram-negative outer membrane.

**TABLE 5:** Lipid peroxidation activity for each potent molecule at MIC values.

Sample No	Lipid peroxidation efficiency (malondialdehyde, nmol mL^–1^)	
	*Achromobacter xylosoxidans* (Gram-negative)	*Enterococcus faecalis* (Gram-positive)
6a	6.352 ± 0.08	7.673 ± 0.26
6c	5.252 ± 0.33	8.815 ± 0.18
Ciprofloxacin	7.724 ± 0.51	9.07 ± 0.32
Control	2.145 ± 0.08	1.664 ± 0.01

#### 2.2.4 DNA gyrase inhibitory activity

An assay to evaluate the inhibition of *E. coli* DNA gyrase was conducted for the two most potent compounds, 6a and 6c, with Ciprofloxacin serving as a reference control. The findings are presented in Table 6, indicating the IC_50_ values in μg/mL. Compound 6a exhibited notable inhibitory activity against DNA gyrase, with an IC_50_ value of 23.75 μg/mL, relative to the Ciprofloxacin control, which recorded an IC_50_ of 15.95 μg/mL. Conversely, compound 6c demonstrated a comparatively lower level of inhibition, being approximately 2.5 times less effective than the control.

**TABLE 6:** Inhibitory activities of the most active compounds (6a and 6c) against E.*Coli* DNA gyrase (supercoiling).

Compound	IC_50_ (µg/mL)
6a	23.75 ± 0.77
6c	40.87 ± 1.33
Ciprofloxacin	15.95 ± 0.52

### 2.3 Molecular modeling simulation

#### 2.3.1 Molecular docking of the tested compounds against DNA Gyrase B

**FIGURE 2:**
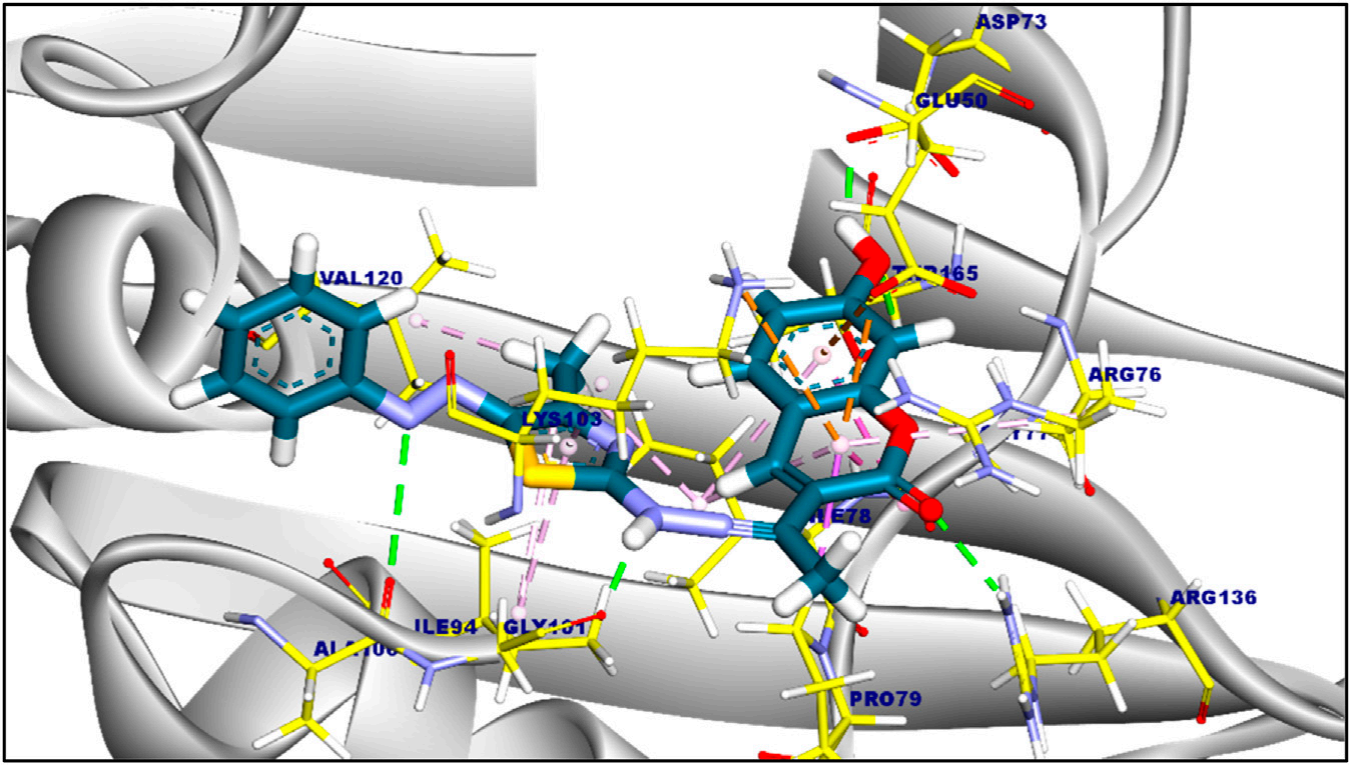
3D figure of the proposed binding mode of compound 6a against DNA Gyrase B. The tested compound is colored by (Blue), and amnio acid side chain by (yellow).

**FIGURE 3:**
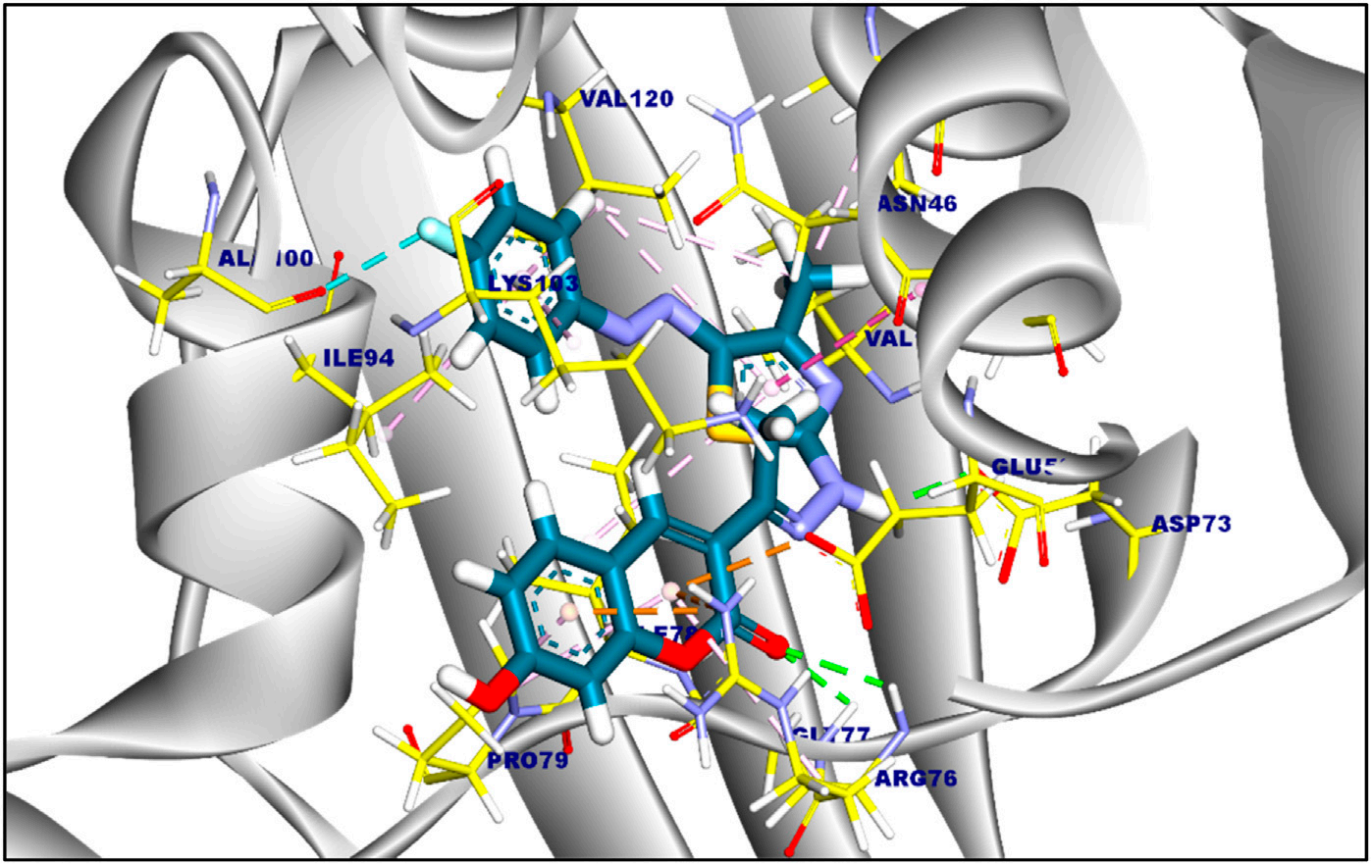
3D figure of the proposed binding mode of compound 6c against DNA Gyrase B. The tested compound is colored by (Blue), and amnio acid side chain by (yellow).

**FIGURE 4:**
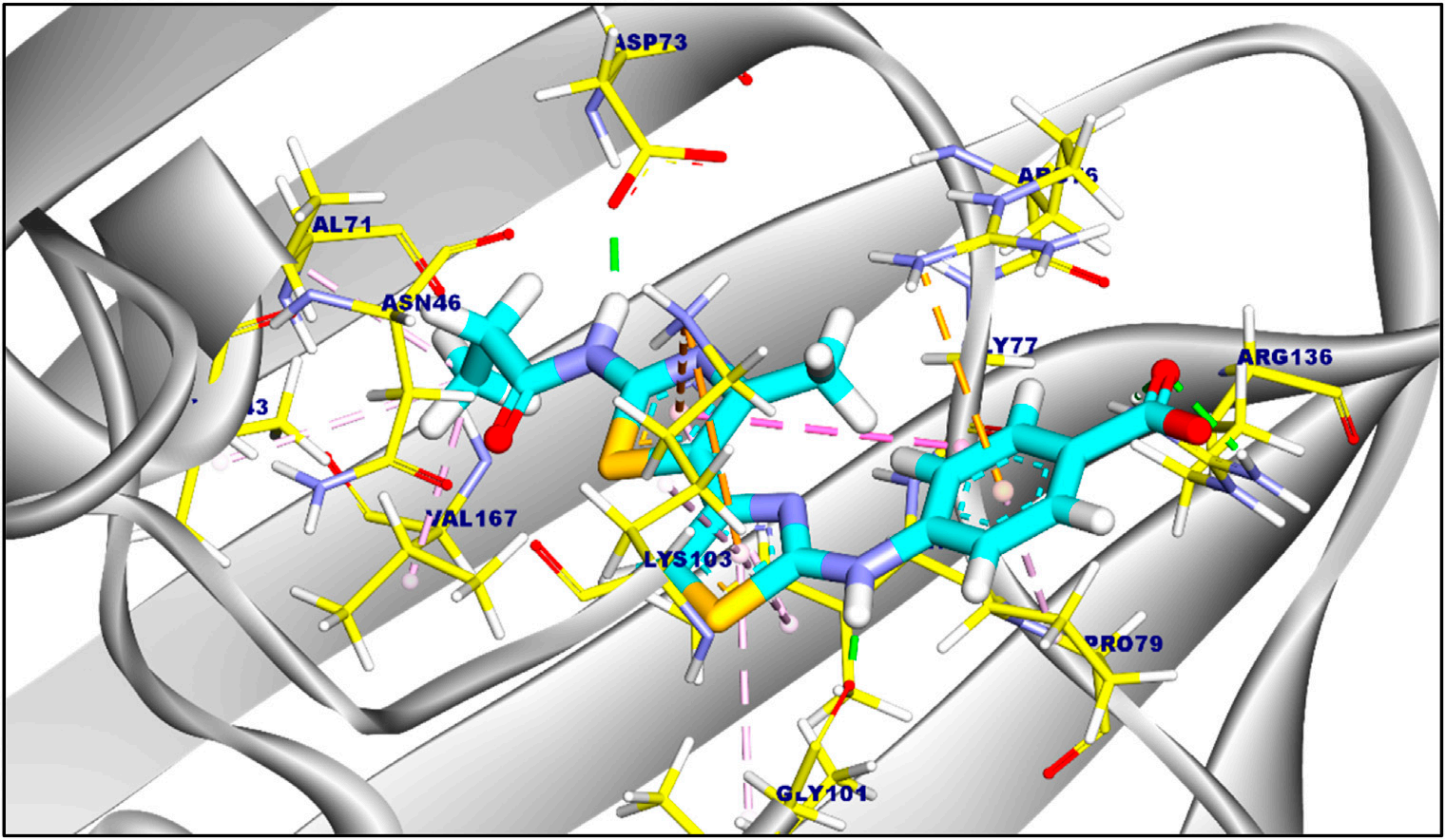
the proposed binding mode of the co-crystalized ligand complexed with DNA Gyrase (B). Ligand is colored by (turquoise), and amnio acid side chain colored by (yellow).

The suggested binding mode of compound 6a demonstrated a binding energy of −8.35 kcal/mol in relation to DNA Gyrase B (PDB ID: 4duh). A total of fifteen interactions were identified, including hydrophobic π-π, π-cation, π-anion, π-alkyl, and π-sigma interactions with Val120, Ile94, Lys103, Ile78, Gly77, Glu50, Pro50, and Arg76 amino acids sidechain. Moreover, compound 6a formed five hydrogen bonds with Ala100, Gly101, Asp73, Thr165, and Arg136 with a distance of 3.29, 1.76, 1.82, 2.37, and 2.42 Å, Figure 2. The binding mode of compound 6c showed a binding energy that was not constrained (∆G) equal of −7.14 kcal/mol. fourteen hydrophobic π-π, π-anion, π-cation, and π-alkyl interactions were conducted with Pro79, Arg76, Glu50, Lys103, Ile94, Val120, Val43, Val167, Ile78, and Asn46. Additionally, the interaction of Compound 6c was stabilized by the formation of three hydrogen bonds with Arg76, Gly77, and Asp73 with distances of 2.59, 2.50, and 2.05 Å (Figure 3). Furthermore, The co-crystalized ligand complexed with DNA Gyrase B exhibited an affinity score of −8.12 kcal/mol with RMSD value equal 1.12 Å, the co-crystalized ligand formed twelve hydrophobic π-alkyl, π-cation, and π-π interactions with Val167, Val43, Val71, Asn46, Gly77, Ile78, Lys103, Ile94, Pro79, and Arg76, additionally, the interaction was supported by three hydrogen bonds with Asp73, Arg136, and Gly101 with distances of 1.87, 2.19 and 1.81 Å (Figure 4).

#### 2.3.2 Molecular dynamic (MD) simulation study for ligand 6a

Molecular dynamics (MD) simulations were performed over duration of 100 nanoseconds to investigate the molecular stability of the ligand 6a, which exhibited the most favorable docking results, in relation to the DNA Gyrase B target pocket. The resulting root mean square deviation (RMSD) values for both the complex and the ligand, in comparison to their initial positions within the active site, were documented and subjected to analysis. Additionally, the interactions of the lead compound 6a were thoroughly examined and assessed (Table 7).

**TABLE 7:** Molecular Docking Analysis of the tested ligands 6a and 6c against DNA Gyrase B.

Compounds	RMSD value (Å)	Docking (affinity) score (kcal/mol)
6a	1.54	−8.35
6c	1.67	−7.14
The co-crystalized ligand	1.15	−8.12

##### 2.3.2.1 Analysis of root mean square deviation (RMSD) and root mean square fluctuation (RMSF) for proteins and ligands

**FIGURE 5:**
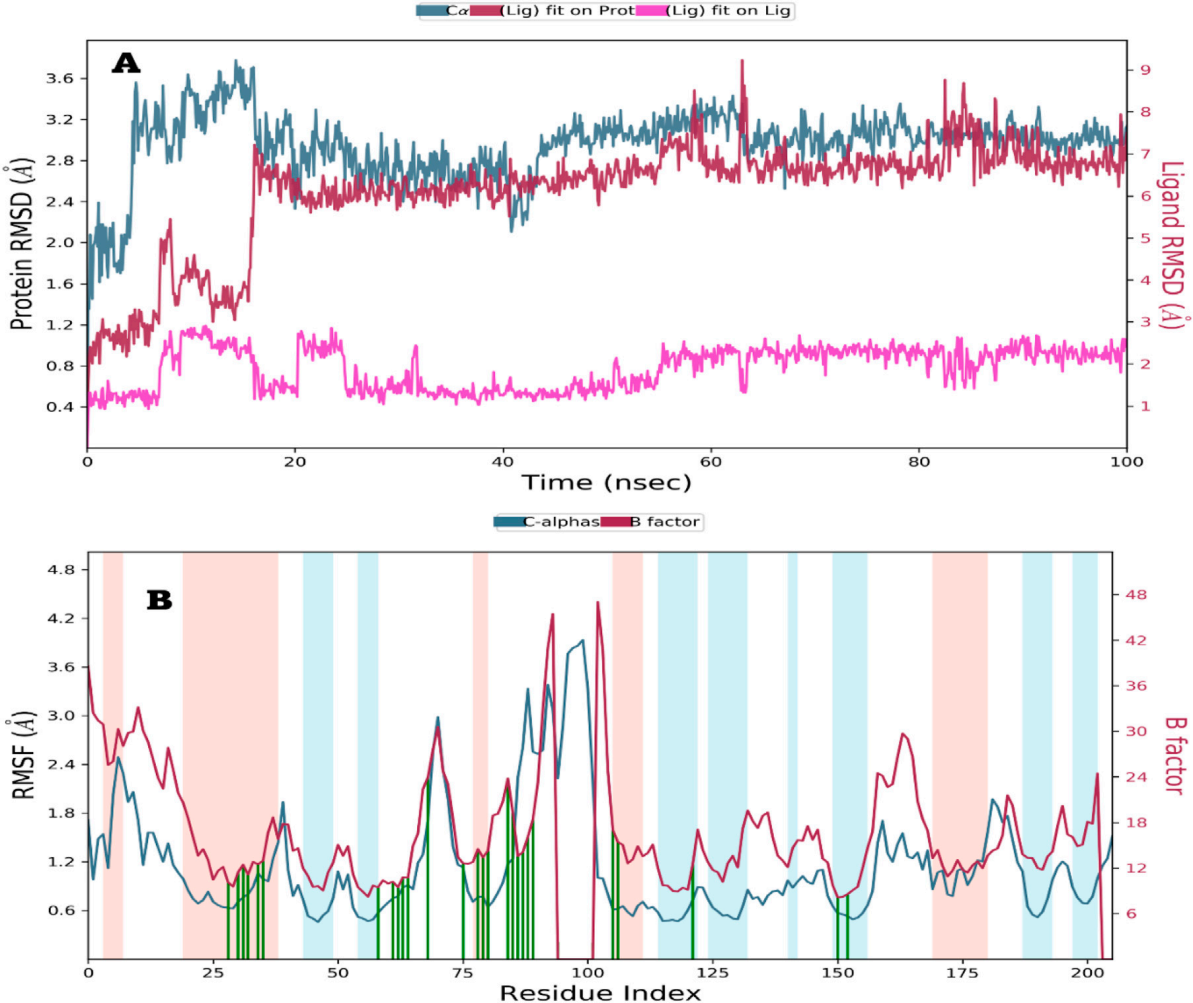
Showed the RMSD and RMSF of Compound 6a against DNA Gyrase B over 100 ns.

Following molecular docking, ligand 6a, which formed a complex with DNA Gyrase B, was chosen for molecular dynamics (MD) simulation. The conformational stability of the protein structure was assessed by tracking the C*α* atoms (indicated by the blue line) in relation to their initial positions. As illustrated in Figure 5A, ligand 6a demonstrated stability within the binding pocket of DNA Gyrase B through the simulation time with fluctuation at 10–20 ns, at this time, target protein loops exhibited many movements at 60–65, 80–110, and 155–170 amino acids areas, which leads to many conformational changes in protein skeleton, that can affect the protein ligand interactions especially at 60–65 amino acids area. Additionally, the protein ligand complex showed stability over the simulation time (20–100 ns) which the ligand reaches equilibrium and fluctuated at 6–7 Å (within RMSD value = 1 Å) (Figure 5B).

##### 2.3.2.2 Analysis of protein-ligand interactions

###### 2.3.2.2.1 Analysis of protein-ligand interaction histograms

**FIGURE 6:**
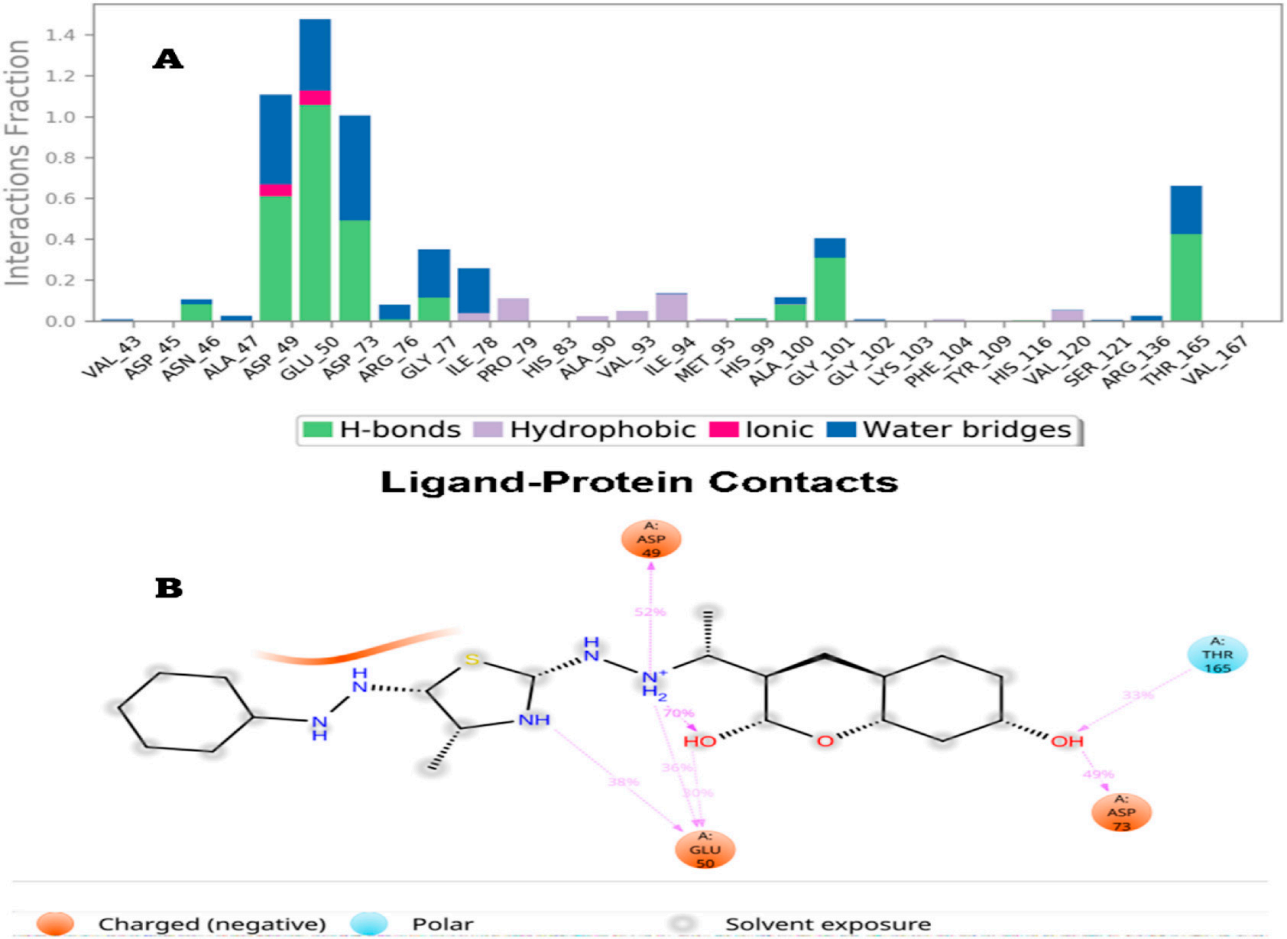
Histogram analysis describing the binding interactions of ligand 6a with DNA Gyrase B during the simulation time (100 ns).

Compound 6a demonstrated significant stability when interacting with DNA Gyrase B, with numerous interactions identified between the compound and the amino acids within the target pocket, which are elaborated upon in detail. Ligand 6a established π-interactions with the subsequent residues: Ile78 (∼5%), Pro79 (∼10%), Val93 (∼5%), and Ile94 (∼20%), As illustrated in Figure 6. Additionally, Ligand 6a exhibited interactions with DNA Gyrase B through multiple polar hydrogen bonds and water-mediated hydrogen bonds. The presence of crystallization water molecules facilitated connections between the protein residues and the ligands, as demonstrated by the specific residues involved, Asn46 (∼15%), Asp49 (∼60%), Glu50 (∼110%), Asp73 (∼50%), Ala100 (∼10%), Gly101 (∼35%), and Thr165 (∼50%). Moreover, Ligand 6a demonstrated the capacity to establish ionic interactions with the residues Asp49 and Glu50.

**FIGURE 7:**
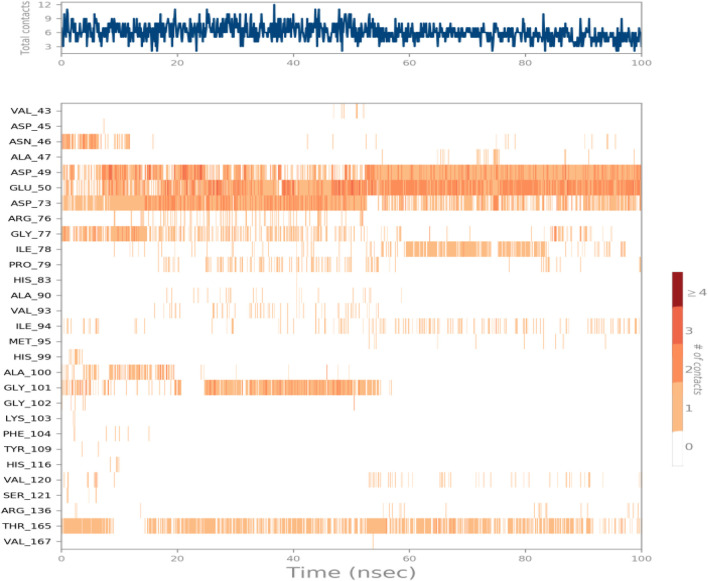
Heat map describing the total interactions within ligand 6a with DNA Gyrase B during the simulation time.

Another method used to monitor these interactions involves plotting the number of interactions concerning time, a heat map Figure 7 indicates the number of interactions at each frame of Compound 6a with DNA Gyrase B, whereas the dark color indicates more interactions. From the heat maps figures, it was observed that the highest number of conformations of the protein of DNA Gyrase B formed up to nine interactions, the most interacted amino acids of DNA Gyrase B with ligand 6a are Asp49, Glu50, Asp73, Gly77, Ile78, Ile94, Gly101 and Thr165.

## 3 Conclusion

The synthesized coumarin-thiazole derivatives showed variable antibacterial activity against drug-resistant pathogens. While ineffective against *Campylobacter jejuni* and *Serratia fonticola*, compounds 6a and 6c exhibited strong activity against *Enterococcus faecalis* and *Achromobacter xylosoxidans* (MICs: 12.5–50 μg/mL). These compounds also inhibited biofilm formation (>50% in *E. faecalis*), induced lipid peroxidation and ROS production, and disrupted bacterial cell integrity. 6a acted as a DNA gyrase inhibitor (IC_50_: 23.75 μg/mL). Molecular docking and dynamics simulations confirmed stable binding of 6a/6c to DNA gyrase, mimicking known inhibitors. These findings highlight 6a and 6c as promising candidates against resistant bacteria.

## 4 Materials and methods

### 4.1 Chemistry

Melting points were determined on electrothermal IA 9000 apparatus and were uncorrected. The NMR analysis including ^1^H and ^13^C-NMR, spectra were recorded using Bruker 500 NMR (Japan) spectrometers (MA, United States) in Faculty of Pharmaceutical Science, Tokushima Bunri University, Japan. The chemical shifts were given in *δ* (ppm), and coupling constants were reported in Hz. Mass spectrum (Direct Inlet part to mass analyzer in Thermo Scientific GCMS model ISQ) and Elemental analyses (FLASH 2000 CHNS/O analyzer, Thermo Scientific (United States) were carried out at the Regional Center for Mycology and Biotechnology (RCMB), Al-Azhar University, Nasr City, Cairo. The reactions were followed by TLC (silica gel, aluminum sheets 60 F254, Merck) using chloroform/methanol (9.8:0.2 v/v) as eluent and sprayed with iodine-potassium. Compounds 3, 4 and 5a-i were previously prepared (Ebaid et al., 2025; Osman et al., 2023).

#### 4.1.1 General procedure for the preparation of target compounds 6a-i

A reaction was performed utilizing a combination of thiosemicarbazones 4 (0.001 mol) and their corresponding hydrazonoyl chloride derivatives 5a-i (0.001 mol) (These derivatives were synthesized using established methods that involve the reaction of diazonium chloride with appropriately activated halogenated methylene groups) in 15 mL of dioxane, with triethylamine (TEA) employed as a catalytic agent. The reaction mixture underwent reflux for a period of 8 h. Following the cooling phase, the resulting precipitate was filtered and subsequently recrystallized from ethanol, leading to the synthesis of the desired compounds 6a-i.

##### 4.1.1.1 7-Hydroxy-3-(-1-{2-[4-methyl-5-(phenyldiazenyl)thiazol-2-yl]hydrazono}ethyl)-2*H*-chromen-2-one (6a)

Red powder, m. p. 228 °C–230 °C, yield (78%). ^1^H NMR (500 MHz, DMSO-d_6_): δ = 2.06 (s, 3H, CH_3_), 2.35 (s, 3H, CH_3_-thiazole), 6.69 (s, 1H, H-Ar), 6.77 (d, 1H, *J* = 8.5 Hz, H-Ar), 7.08-7.14 (m, 1H, H-Ar), 7.37 (d, 2H, *J* = 8.5 Hz, H-Ar), 7.58 (d, 1H, *J* = 8.00 Hz, H-Ar), 7.74 (d, 2H, *J* = 8.5 Hz, 1H, H-Ar), 7.76 (d, 1H, *J* = 1.5 Hz, H_4_ of coumarin), 10.54 (s, 1H, NH), 10.72 (s, 1H, OH). ^13^C NMR (126 MHz, DMSO) δ = 14.25 (CH_3_), 15.49 (CH_3_), 104.10, 119.37, 121.03, 121.44, 125.07, 125.77, 128.25, 128.66, 129.75, 132.21, 134.58, 138.63, 147.28, 158.51, 159.60(C-OH), 160.64(C=O), 171.50(C=N). Analysis for C_21_H_17_N_5_O_3_S (419.45), Calcd.: % C, 60.13; H, 4.09; N, 16.70; Found: % C, 60.24; H, 4.21; N, 16.82. MS m/z (R.A.%): 419 (M^+^) (34.33%), 368 (100.00%).

##### 4.1.1.2 7-Hydroxy-3-(-1-{2-[4-methyl-5-(*p*-tolyldiazenyl)thiazol-2-yl]hydrazono}ethyl)-2*H*-chromen-2-one (6b)

Brown powder, m. p. 197 °C–199 °C, yield (85%). ^1^H NMR (500 MHz, DMSO-d_6_): δ = 2.29 (s, 3H, CH_3_), 2.35 (s, 3H, CH_3_), 2.45 (s, 3H, CH_3_-thiazole), 6.95 (s, 1H, H-Ar), 7.20 (d, 1H, *J* = 8.0 Hz, H-Ar), 7.30 (d, 1H, *J* = 8.5 Hz, H-Ar), 7.68 (d, 2H, *J* = 8.5 Hz, H-Ar), 7.83 (s, 1H, H_4_ of coumarin), 7.87 (d, 2H, *J* = 8.5 Hz, 1H, H-Ar), 10.12 (s, 1H, NH), 10.91 (s, 1H, OH). ^13^C NMR (126 MHz, DMSO) δ = 14.54 (CH_3_), 16.05 (CH_3_), 20.00 (CH_3_), 101.82, 106.22, 113.81, 121.76, 125.53, 125.97, 128.82, 130.07, 131.98, 137.33, 141.89, 150.89, 154.45, 155.89, 155.97, 161.26 (C=O), 171.19(C=N). Analysis for C_22_H_19_N_5_O_3_S (433.48), Calcd.: % C, 60.96; H, 4.42; N, 16.16;. Found: % C, 61.06; H, 4.54; N, 16.25. MS m/z (R.A.%): 433 (M^+^) (19.01%), 81 (100.00%).

##### 4.1.1.3 3-(-1-{2-[5-((4-fluorophenyl)diazenyl)-4-methylthiazol-2-yl]hydrazono}ethyl)-7-hydroxy-2*H*-chromen-2-one (6c)

Red powder, m. p. 207 °C–209 °C, yield (77%). ^1^H NMR (500 MHz, DMSO-d_6_): δ = 2.26 (s, 3H, CH_3_), 2.41 (s, 3H, CH_3_-thiazole), 6.88 (s, 1H, H-Ar), 7.00 (d, 1H, *J* = 8.0 Hz, H-Ar), 7.42 (d, 2H, *J* = 8.5 Hz, H-Ar), 7.59 (d, 1H, *J* = 8.0 Hz, H-Ar), 7.80 (d, 2H, *J* = 8.5 Hz, H-Ar), 7.98 (s, 1H, H_4_ of coumarin), 10.25 (s, 1H, NH), 11.15 (s, 1H, OH). ^13^C NMR (126 MHz, DMSO) δ = 14.16 (CH_3_), 17.65(CH_3_), 101.82, 106.22, 113.81, 125.53, 125.97, 128.84, 130.07, 131.98, 137.33, 141.80, 141.89, 150.62, 154.51(C-OH), 155.97(C9), 159.21(C=O), 161.26(C-F), 171.94(C=N). Analysis for C_21_H_16_FN_5_O_3_S (437.44), Calcd.: % C, 57.66; H, 3.69; N, 16.01. Found: % C, 57.77; H, 3.82; N, 16.13. MS m/z (R.A.%): 437 (M^+^) (18.75%), 225 (100.00%).

##### 4.1.1.4 3-(1-{2-[5-(4-Bromophenyl)diazinyl]-4-methylthiazol-2-yl}hydrazono)ethyl-7-hyd- roxy-2*H*-chromen-2-one (6d)

Red powder, m. p. 215 °C–217 °C, yield (88%). ^1^H NMR (500 MHz, DMSO-d_6_): δ = 2.29 (s, 3H, CH_3_), 2.35 (s, 3H, CH_3_-thiazole), 6.72 (d, 1H, *J* = 8.0 Hz, H-Ar), 6.93 (s, 1H, H-Ar), 7.53 (d, 1H, *J* = 8.5 Hz, H-Ar), 7.68 (d, 2H, *J* = 8.0 Hz, H-Ar), 7.84 (s, 1H, H4 of coumarin), 7.87 (d, 2H, *J* = 8.0 Hz, H-Ar), 10.32 (s, 1H, NH), 11.33 (s, 1H, OH). ^13^C NMR (126 MHz, DMSO) δ = 13.09 (CH_3_), 15.75(CH_3_), 101.70, 111.09, 111.64, 113.44, 113.70, 118.64, 120.45, 121.52, 124.08, 129.97, 130.29, 130.37, 142.79, 155.71, 156.25, 160.71(C=O), 172.20 (C=N). Analysis for C_21_H_16_BrN_5_O_3_S (498.35), Calcd.: % C, 50.61; H, 3.24; N, 14.05. Found: % C, 50.73; H, 3.33; N, 14.16. MS m/z (R.A.%): 498 (M^+^) (39.46%), 163 (100.00%).

##### 4.1.1.5 7-Hydroxy-3-(1-{2-[5-(4-methoxyphenyl)diazinyl]-4-methylthiazol-2-yl}hydrazono) ethyl-2*H*-chromen-2-one (6e)

Red powder, m. p. 226 °C–227 °C, yield (91%). ^1^H NMR (500 MHz, CDCl_3_): δ = 2.23 (s, 3H, CH_3_), 3.33 (s, 3H, CH_3_-thiazole), 3.85 (s, 3H, OCH_3_), 6.74 (s, 1H, H-Ar), 6.80 (d, 1H, *J* = 8.0 Hz, H-Ar), 6.94 (d, 2H, *J* = 8.5 Hz, H-Ar), 7.44 (d, 1H, *J* = 8.0 Hz, H-Ar), 7.70 (d, 2H, *J* = 8.5 Hz, H-Ar), 8.00 (s, 1H, H4 of coumarin), 10.13 (s, 1H, NH), 10.97 (s, 1H, OH). ^13^C NMR (126 MHz, CDCl_3_) δ 14.13 (CH_3_), 14.96 (CH_3_), 55.37 (OCH_3_), 102.58, 112.31, 113.61, 113.91, 114.08, 119.17, 119.64, 122.07, 127.21, 130.08, 132.05, 144.78, 155.95, 159.44 (C-OH), 160.93(C=O), 161.39(C-OCH_3_), 172.05 (C=N). Analysis for C_22_H_19_N_5_O_4_S (449.48), Calcd.: % C, 58.79; H, 4.26; N, 15.58. Found: % C, 58.91; H, 4.33; N, 15.67. MS m/z (R.A.%): 449 (M^+^) (29.80%), 62 (100.00%).

##### 4.1.1.6 3-(1-{2-[5-(2,4-Dichlorophenyl)diazinyl]-4-methylthiazol-2-yl}hydrazono)ethyl-7-hydroxy-2*H*-chromen-2-one (6f)

Red powder, m. p. 194 °C–196 °C, yield (89%). ^1^H NMR (500 MHz, DMSO-d_6_): δ = 2.29 (s, 3H, CH_3_), 2.46 (s, 3H, CH_3_-thiazole), 6.95 (s, 1H, H-Ar), 7.17-7.21 (m, 2H, H-Ar), 7.32 (s, 1H, H-Ar), 7.53 (d, 1H, *J* = 8.5 Hz, H-Ar), 7.69 (d, 1H, *J* = 8.0 Hz, H-Ar), 8.05 (s, 1H, H4 of coumarin), 10.06 (s, 1H, NH), 10.97 (s, 1H, OH). ^13^C NMR (126 MHz, DMSO), δ = 13.06 (CH_3_), 14.31(CH_3_), 101.69, 101.90, 102.20, 107.79, 111.02, 111.77, 113.73, 114.97, 121.10, 130.35, 132.23, 142.95, 147.41, 148.40, 156.06, 158.89, 160.86(C-OH), 162.64(C=O), 170.31(C=N). Analysis for C_21_H_15_C_l2_N_5_O_3_S (488.34), Calcd.: % C, 51.65; H, 3.10; N, 14.34. Found: % C, 51.76; H, 3.21; N, 14.44. MS m/z (R.A.%): 488 (M^+^) (22.92%), 240 (100.00%).

##### 4.1.1.7 3-(1-{2-[5-(4-Acetylphenyl)diazinyl]-4-methylthiazol-2-yl}hydrazono)ethyl-7-hydroxy-2*H*-chromen-2-one (6g)

Red powder, m. p. 205 °C–207 °C, yield (76%). ^1^H NMR (500 MHz, DMSO-d_6_): δ = 2.29 (s, 3H, CH_3_), 2.34 (s, 3H, CH_3_-thiazole), 2.46 (s, 3H, COCH_3_), 6.72 (d, 1H, *J* = 8.0 Hz, H-Ar), 7.32 (s, 1H, H-Ar), 7.54 (d, 1H, *J* = 8.5 Hz, H-Ar), 7.68 (d, 2H, *J* = 8.0 Hz, H-Ar), 7.83 (s, 1H, H4 of coumarin), 7.87 (d, 2H, *J* = 8.0 Hz, H-Ar), 10.22 (s, 1H, NH), 11.16 (s, 1H, OH). ^13^C NMR (126 MHz, DMSO) δ = 14.31(CH_3_), 16.05 (CH_3_-thiazole), 22.38 (CH_3_-CO), 101.69, 107.79, 111.77, 113.73, 114.97, 121.10, 130.35, 132.23, 142.95, 148.40, 156.06, 158.89, 160.86, 161.10, 162.64 (C2 of coumarin), 171.94(C=N), 196.13 (C=O). Analysis for C_23_H_19_N_5_O_4_S (461.49), Calcd.: % C, 59.86; H, 4.15; N, 15.18;. Found: % C, 59.96; H, 4.24; N, 15.30. MS m/z (R.A.%): 461 (M^+^) (17.32%), 191 (100.00%).

##### 4.1.1.8 3-(1-{2-[5-(2-chloro-4-nitrophenyl)diazinyl]-4-methylthiazol-2-yl}hydrazono) ethyl-7-hydroxy-2*H*-chromen-2-one (6h)

Brown powder, m. p. 237 °C–239 °C, yield (88%). ^1^H NMR (500 MHz, DMSO-d_6_): δ = 2.18 (s, 3H, CH_3_), 2.41 (s, 3H, CH_3_-thiazole), 6.29 (s, 1H, H-Ar), 6.92 (d, 1H, *J* = 8.0 Hz, H-Ar), 7.26 (d, 1H, *J* = 7.5 Hz, H-Ar), 7.44 (d, 1H, *J* = 8.0 Hz, H-Ar), 7.59 (s, 1H, H-Ar), 7.88 (d, 1H, *J* = 8.0 Hz, H-Ar), 8.30 (s, 1H, H-Ar), 10.11 (s, 1H, NH), 11.41 (s, 1H, OH). ^13^C NMR (126 MHz, DMSO) δ = 13.09 (CH_3_), 15.75 (CH_3_-thiazole), 101.70, 111.09, 111.64, 113.70, 113.44, 118.64, 120.45, 121.52, 129.97, 130.29, 130.37, 142.79, 156.25, 159.08, 160.37, 160.71, 160.78 (C2 of coumarin), 172.20 (C=N). Analysis for C_21_H_15_ClN_6_O_5_S (498.89), Calcd.: % C, 50.56; H, 3.03; N, 16.85; Found: C, 50.67; H, 3.14; N, 16.95. MS m/z (R.A.%): 498 (M^+^) (35.06%), 148 (100.00%).

##### 4.1.1.9 4-(2-{2-[1-(7-Hydroxy-2-oxo-2*H*-chromen-3-yl)ethylidene]hydrazinyl}-4-methyl thiazol-5-yl) diazinyl benzenesulfonamide (6i)

Red powder, m. p. > 300 °C, yield (74%). ^1^H NMR (500 MHz, DMSO-d_6_): δ = 2.40 (s, 3H, CH_3_), 2.47 (s, 3H, CH_3_-thiazole), 6.74 (s, 1H, H-Ar), 6.81 (d, 1H, *J* = 8.5 Hz, H-Ar), 6.94 (d, 2H, *J* = 7.0 Hz, H-Ar), 7.26-7.31 (m, 4H, H-Ar, NH_2_), 7.63 (d, 1H, *J* = 8.5 Hz, H-Ar), 8.24 (s, 1H, H4 of coumarin), 10.56 (s, 1H, NH), 10.84 (s, 1H, OH). ^13^C NMR (126 MHz, DMSO) δ = 14.26 (CH_3_), 15.52(CH_3_), 103.28, 119.09, 121.06, 121.52, 125.04, 125.67, 127.94, 128.26, 128.69, 129.62, 133.12, 134.61, 141.87, 147.92, 149.81, 152.23, 171.24 (C=N). Analysis for C_21_H_18_N_6_O_5_S_2_ (498.53), Calcd.: % C, 50.59; H, 3.64; N, 16.86; Found: C, 50.70; H, 3.72; N, 16.95. MS m/z (R.A.%): 498 (M^+^) (18.83%), 366 (100.00%).

### 4.2 Biological activity

#### 4.2.1 Antibacterial activity of the prepared coumarin derivatives

To assess the antibacterial effectiveness of the synthesized coumarin derivatives, various extensively drug-resistant (XDR) bacterial pathogens were employed. In this regard, two Gram-positive (*Campylobacter jejuni* and *Enterococcus faecalis*) and two Gram-negative (*Serratia fonticola* and *Achromobacter xylosoxidans*) bacteria were supplemented by faculty of medicine (Boys), Al-Azhar University. Antibiogram sensitivity profile demonstrated highly resistance of these pathogens towards at least three groups of classical antibiotic references. Activation of each of pathogen prior to screening of the targeted compounds was implemented Cultivating in Mueller Hinton broth for 24 h at 37 °C with shaking. The rationale for the inoculum size for each pathogen was determined through the use of the McFarland scale and precisely quantified in terms of colony forming units (CFU/mL) to ensure a constant bacterial concentration across all experimental assays. Thus, The agar-well diffusion method was developed in accordance with the guidelines established by the Clinical and Laboratory Standards Institute (CLSI) (Yassin et al., 2025). Subsequently, screening of each of targeted molecule was performed at fixed concentration (50 μg/mL). Incubation of each tested pathogen was carried out and the observed inhibition zone diameter (mm) were conducted in triplicate and subsequently compared to established standard antibacterial agents (Ahmed et al., 2024).

#### 4.2.2 Determination of MIC values

The most-active compounds that efficiently inhibited the tested bacterial pathogens were subjected to determine the MIC using broth microdilution procedure according to CLSI protocol. Briefly, a predetermined quantity, 10 mg of each compound and standard drug was solubilized in 1 mL of dimethyl sulphoxide (DMSO) to create a stock solution. Ten successive dilutions were prepared with a concentration 10 times greater than the final solution of the targeted compounds. The dilutions were further diluted to 1:5 in Mueller Hinton broth medium and 100 µL aliquots were sequentially dispensed into the microdilution plates to obtain the desired concentrations ranging from 20 to 250 μg/mL. In comparison, the standard antibacterial drug was also prepared as same as the targeted compounds and determined its MIC value. After incubation period, 80 µL of each microbial cell was swapped onto nutrient agar plates. The determination of the minimum inhibitory concentration (MIC) for each targeted compound is defined as the lowest concentration of each sample that results in a minimum number of colony-forming units (CFUs) when compared to the untreated samples (Mohamed et al., 2023).

Briefly, 10 mg of each selected compound and reference drugs were dissolved in 1 mL of dimethyl sulphoxide (DMSO) to prepare the stock solution. Ten successive dilutions were prepared with a concentration 10 times greater than the final solution of the synthesized compounds. The dilutions were further diluted to 1:5 in PDB and 100 µL aliquots were sequentially dispensed into the microdilution plates to get the desired concentrations ranging from10–400 μg/mL. Each tested compound was incubated with the tested fungal strain for 48 h at 28 °C. The PDB mixed with DMSO was served as the control growth samples. After that, inoculated of each treated sample with 50 µL to Potato dextrose agar plate and incubated for 72 h at 28 ^o^C

#### 4.2.3 Biofilm inhibitory activity

##### 4.2.3.1 Qualitative method using Congo red agar plate

The Congo Red Agar (CRA) method was employed to qualitatively evaluate biofilm formation in bacteria subjected to a fixed concentration of polymer (20 μg/mL). The CRA medium was formulated with brain heart infusion broth (37 g/L), sucrose (50 g/L), agar (10 g/L), and Congo red indicator (0.8 g/L). Bacterial cultures that had been treated with the polymer were inoculated onto CRA plates and incubated aerobically at 37 °C for a duration of 24 h. The presence of black colonies exhibiting a dry, crystalline morphology indicated successful biofilm production, while red colonies were indicative of inhibited biofilm formation (Ragab et al., 2023).

##### 4.2.3.2 Quantitative method using crystal violet assay

Quantitative evaluation of biofilm inhibition was conducted using crystal violet assay. Anti-biofilm activity of each potent molecule was investigated at MIC value, which mixed with 170 µL Mueller Hinton broth and then inoculated with 15 *µ*L of bacterial pathogen in 96-well plate and incubated at 37 °C for 24 h. The culture medium was removed, and the wells were rinsed with 200 µL of phosphate-buffered saline (PBS) at pH 7.2 to eliminate any planktonic cells, followed by a drying period of 1 hour in a sterilized laminar flow hood. Subsequently, 200 µL of a 0.1% (w/v) crystal violet solution was added to each well and allowed to incubate for 1 hour. Excess stain was then removed by washing the wells three times with PBS, after which the plates were dried. Finally, 200 µL of ethanol was introduced to elute the crystal violet, and the absorbance was measured at 590 nm (Basnet et al., 2023).

The biofilm mass inhibition % was calculated according to the following formula:

$$\text{Biofilm mass inhibition }\left(\%\right)=\left[\left(C-S\right)/C\right]\times 100$$

Where C is the optical density at 590 nm (OD_590_) of the untreated bacterial cells and S is the OD_590_ of the treated bacterial cells.

#### 4.2.4 Measurement of intracellular reactive oxygen species (ROS) inside the treated microbial cells

Reactive Oxygen Species is one of the assumed mechanisms that may responsible for the antibacterial activity of the Sulfa drugs. ROS are known as a group of highly reactive molecules like molecular oxygen (O_2_), superoxide anion (O_2_^•^), hydrogen peroxide (H_2_O_2_) and hydroxyl radicals (OH^•^) having an ability to induced an oxidative stress onto the bacterial cells. Therefore, the susceptibility of the potent compounds to induced ROS toward the microbial pathogens was assessed intracellularly. In this regard, ROS could be quantifying using a fluorescent prop 2′,7′-Dichlorodihydrofluorescein diacetate (DCFH-DA). Briefly, the treated microbial samples were subjected to permeabilization process by ultrasonciation of cells and the resultant supernatant was harvested and reacted with 10 μM DCFH-DA for 30 min at 37 °C (Farid et al., 2024). At the same time, the microbial cells were treated with hydrogen peroxide H_2_O_2_ at 155 μM as a positive control. Immediately, the DCF fluorescence intensity was measured by Spectrofluorometric apparatus (JASCO FP-6500, light source Xenon arc lamp, Japan). The fluorescence intensity for each sample was measured with an excitation and emission wavelength at 485 nm and 530 nm, respectively.

#### 4.2.5 Effect of coumarin derivatives on the bacterial lipid peroxidation (LPO)

A treated bacterial cell using the most potent molecules was subjected to investigate the LPO process. Utilizing the LPO colorimetric kit assay, a particular reagent called thiobarbituric acid (TBA) was utilized to indicate the fatty acid peroxidation byproduct, malondialdehyde (MDA), producing a pink color complex known as MDA-TBA (Mallique Qader et al., 2021). In this study, a consistent concentration of each potent molecule (20 μg/mL) was employed to evaluate the level of oxidation occurring in the lipids of the bacterial cell membrane. To assess lipid peroxidation (LPO), 1 mL of each treated bacterial pathogen was combined with 300 μL of malondialdehyde (MDA) lysis buffer at 4 °C. Subsequently, 3 μL of Butylated Hydroxytoluene (BHT) was added to mitigate interference from pigments generated by the decomposition of lipophilic peroxides. Each sample was then subjected to centrifugation at 8,000 rpm for 10 min, after which the insoluble materials were discarded. A volume of 200 μL of the clear supernatant was mixed with 600 μL of thiobarbituric acid (TBA) solution and incubated at 95 °C for 60 min. Following incubation, the samples were cooled to 25 °C, and the resultant pink color was quantified using a spectrophotometer (Agilent Cary 100, Germany) at a wavelength of 532 nm. Treatment of both bacterial pathogens using 5% hydrogen peroxide for 20 min as a positive control was also involved. The increase in the malondialdehyde concentration in the tested sample indicated the increase of the lipid peroxidation activity of SL towards bacterial cell membrane, malondialdehyde concentration was calculated based on the following equation:

$$\text{Malondialdehyde}\left(\text{nmol}/\text{mL}\right)=A\text{ sample}/A\text{ standard}\times 10\ldots$$

Where A sample is the lipid peroxidation absorbance in the treated bacterial cells and A standard is the absorbance of the standard lipid peroxidation sample.

#### 4.2.6 Enzyme inhibitory effects against *E.Coli* DNA Gyrase

The *in vitro* assessment of enzyme inhibition for the most active derivative 6a and 6c was performed against *E. coli* DNA gyrase B. The anticipated assay was done *using E. coli* DNA gyrase microplate assay kit (Inspiralis) according to the optimized protocol of the manufacturer. Ciprofloxacin was used as a reference drug for *E. coli* DNA gyrase B according to the previously reported methods (Fayed et al., 2022). The obtained data are tabulated as IC_50_ values in Table 6.

### 4.3 Molecular modeling

#### 4.3.1 Molecular docking analysis

Molecular docking studies were conducted to evaluate the potential binding affinity of the compounds 6a and 6c towards DNA Gyrase B. the tested compounds were docked against DNA Gyrase B obtained from Protein Data Bank (PDB ID: 4duh) (Brvar et al., 2012).

Initially, water molecules and extraneous components were eliminated from the protein complex. Subsequently, crystallographic disorders and unoccupied valence atoms were rectified. The energy of the protein structure was minimized and subsequently saved in a PDBQT file format. The two-dimensional structures of each compound were constructed utilizing Chem-Bio Draw Ultra 16.0, saved as SDF files, and subsequently converted into three-dimensional structures. Protonation and energy minimization were performed, with the results also saved as PDBQT files. The docking processes were executed using AutoDock Vina version 1.5.7, employing a rigid docking technique in which the receptor remained fixed while the ligands were permitted to adopt flexible conformations. Furthermore, each molecule was allowed to generate twenty distinct poses. The docking scores, representing the affinity energy of the most favorable poses in relation to the target protein, were documented, and both three-dimensional and two-dimensional representations were produced using Discovery Studio 2024 for visualization purposes (El-Demerdash et al., 2024).

#### 4.3.2 Molecular dynamic (MD) simulation

The Desmond simulation package developed by Schrödinger LLC was employed to perform molecular dynamics (MD) simulations (Kumar et al., 2022). All simulations were conducted using the NPT ensemble, maintaining a temperature of 300 K and a pressure of 1 bar throughout the experimental runs. Each simulation was executed for a duration of 100 ns, with a relaxation time of 1 ps allocated for the ligands under investigation. The OPLS_2005 force field parameters were utilized for all simulations. Long-range electrostatic interactions were calculated using the particle mesh Ewald method, with a cutoff radius of 9.0 Å designated for Coulomb interactions (Ivanova et al., 2018).

Water molecules were represented explicitly through the simple point charge model. Pressure regulation was achieved using the Martyna–Tuckerman–Klein chain coupling scheme, characterized by a coupling constant of 2.0 ps, while temperature regulation was conducted via the Nosé–Hoover chain coupling scheme. Nonbonded interactions were computed utilizing the r-RESPA integrator, wherein short-range forces were updated at every time step and long-range forces were updated every three-time steps. Trajectories were recorded at intervals of 4.8 ps for further analysis.

The interactions and behavior of ligands and proteins were analyzed using the Simulation Interaction Diagram tool available in the Desmond molecular dynamics (MD) package. The stability of the MD simulations was evaluated by tracking the root mean square deviation (RMSD) of the positions of ligand and protein atoms over time (Case et al., 2008).

## Data Availability

The datasets presented in this study can be found in online repositories. The names of the repository/repositories and accession number(s) can be found in the article/Supplementary Material.
