# Supplementary material for: Design, synthesis, and biological assessment of a novel series of coumarin-tethered thiazole derivatives as potential antibacterial agents
Source: Front Chem. 2025 Sep 5;13:1627186. doi: 10.3389/fchem.2025.1627186 (PMC12447640; doi:10.3389/fchem.2025.1627186)
Supplement: Supplementary file 1 [file DataSheet1.pdf]

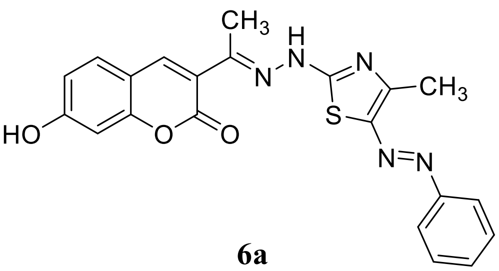
CH3
H
N
N
N
CH3
S
HO
O
O
N
N

6a

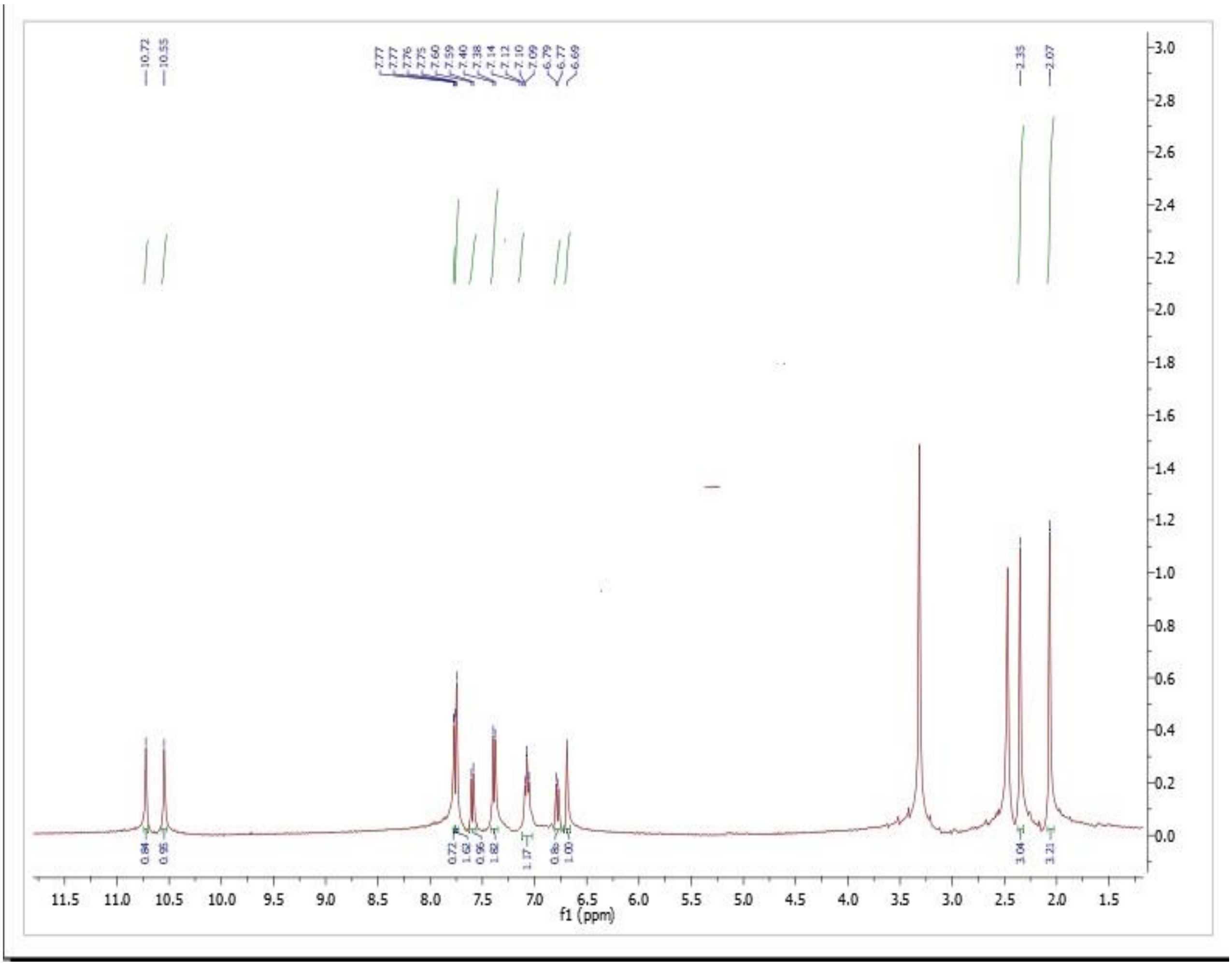
10.72
10.55
7.77
7.77
7.76
7.75
7.60
7.59
7.40
7.38
7.14
7.12
7.10
7.09
6.79
6.77
6.69
2.35
2.07
0.84
0.95
0.72
1.62
0.96
1.82
1.17
0.86
1.00
3.04
3.21
11.5 11.0 10.5 10.0 9.5 9.0 8.5 8.0 7.5 7.0 6.5 6.0 5.5 5.0 4.5 4.0 3.5 3.0 2.5 2.0 1.5
f1 (ppm)
3.0 2.8 2.6 2.4 2.2 2.0 1.8 1.6 1.4 1.2 1.0 0.8 0.6 0.4 0.2 0.0

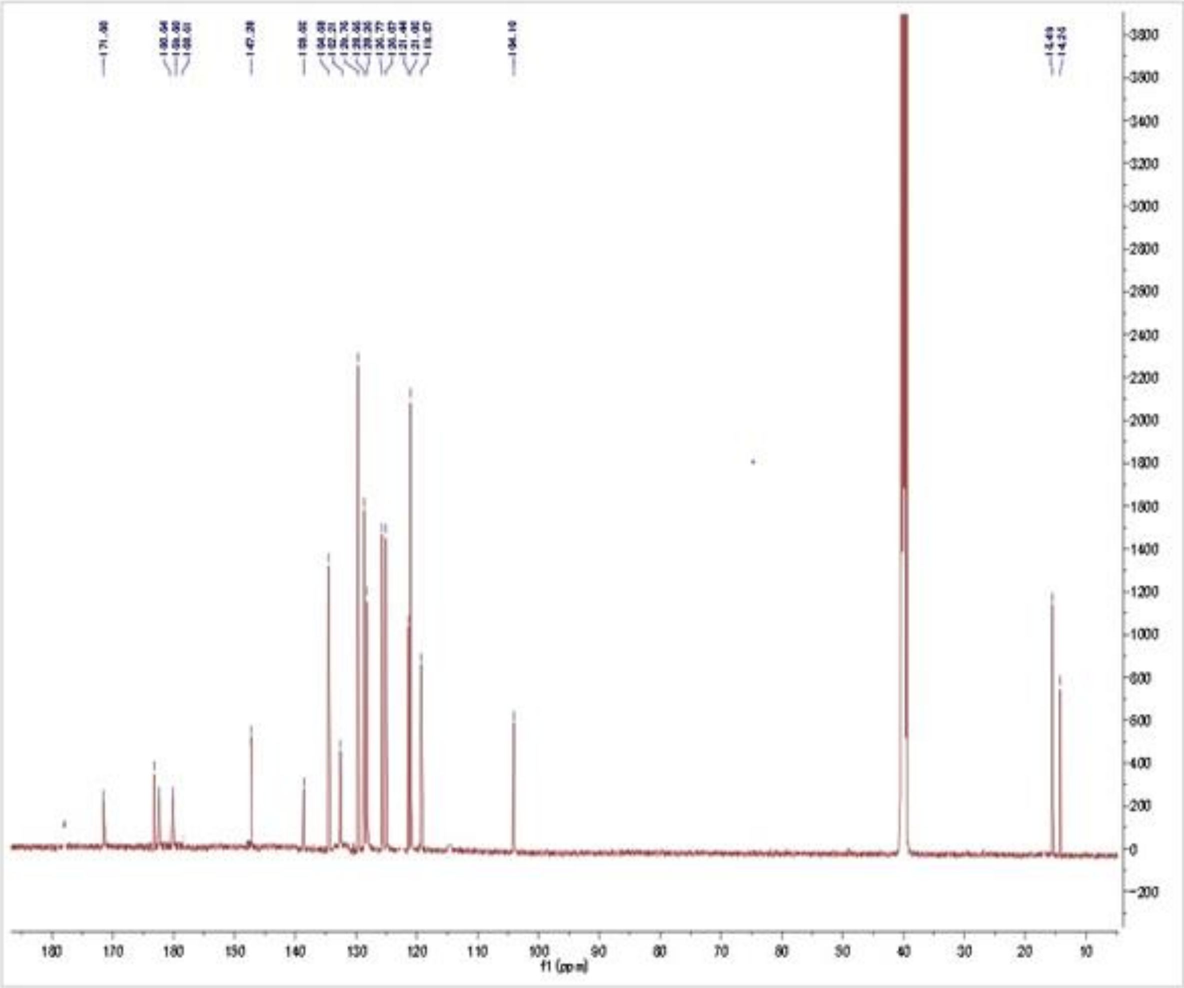
f1 (ppm)

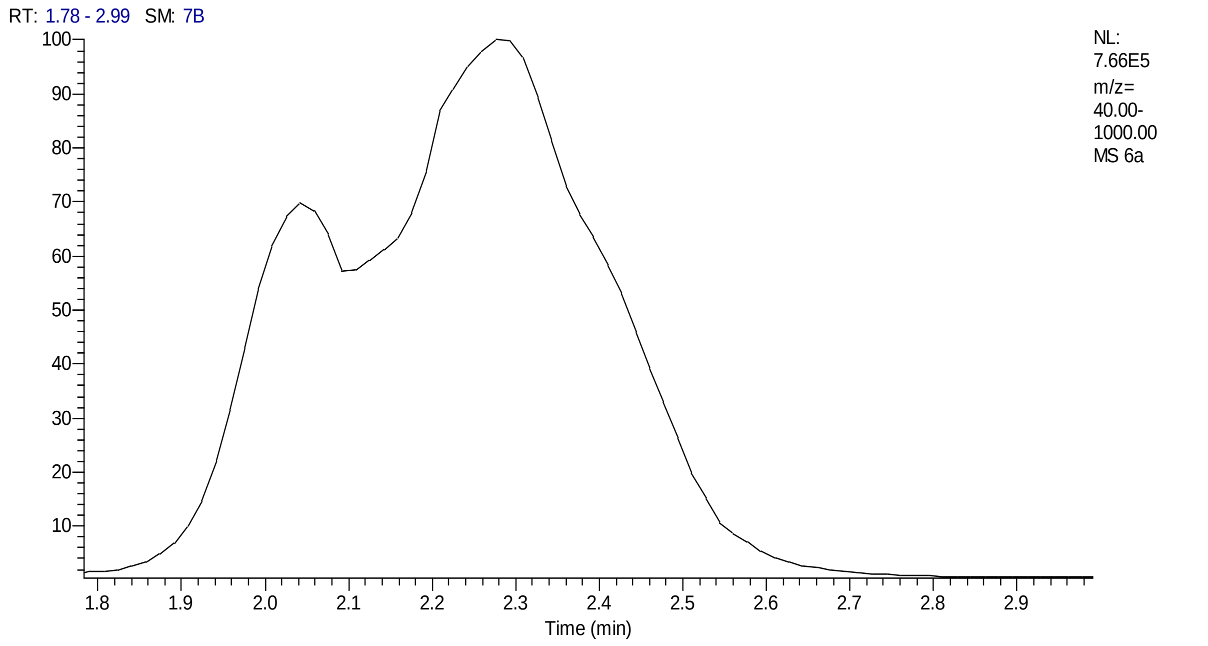

RT: 1.78 - 2.99 SM: 7B
NL: 7.66E5
m/z= 40.00-1000.00
MS 6a
Time (min)

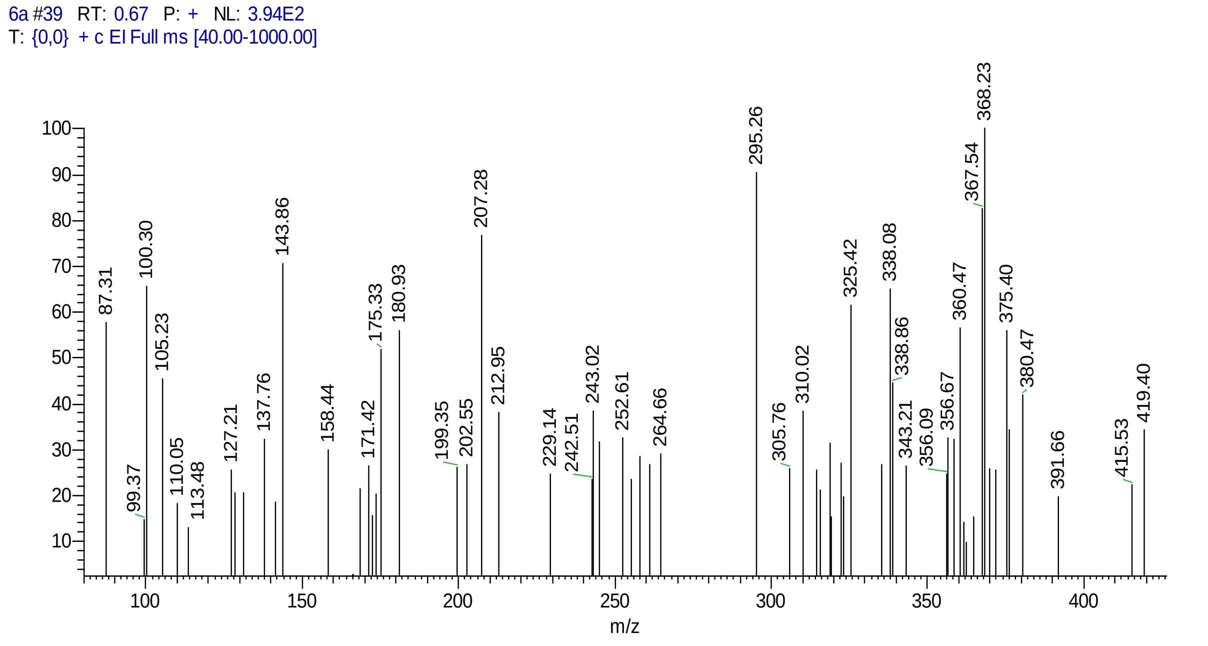

6a #39 RT: 0.67 P: + NL: 3.94E2
T: {0,0} + c EI Full ms [40.00-1000.00]
87.31
99.37
100.30
105.23
110.05
113.48
127.21
137.76
143.86
158.44
171.42
175.33
180.93
199.35
202.55
207.28
212.95
229.14
242.51
243.02
252.61
264.66
295.26
305.76
310.02
325.42
338.08
338.86
343.21
356.09
356.67
360.47
367.54
368.23
375.40
380.47
391.66
415.53
419.40
m/z

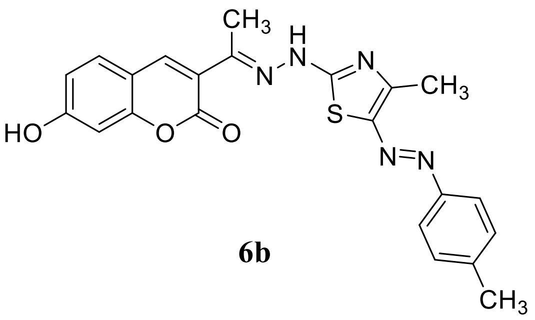

6b

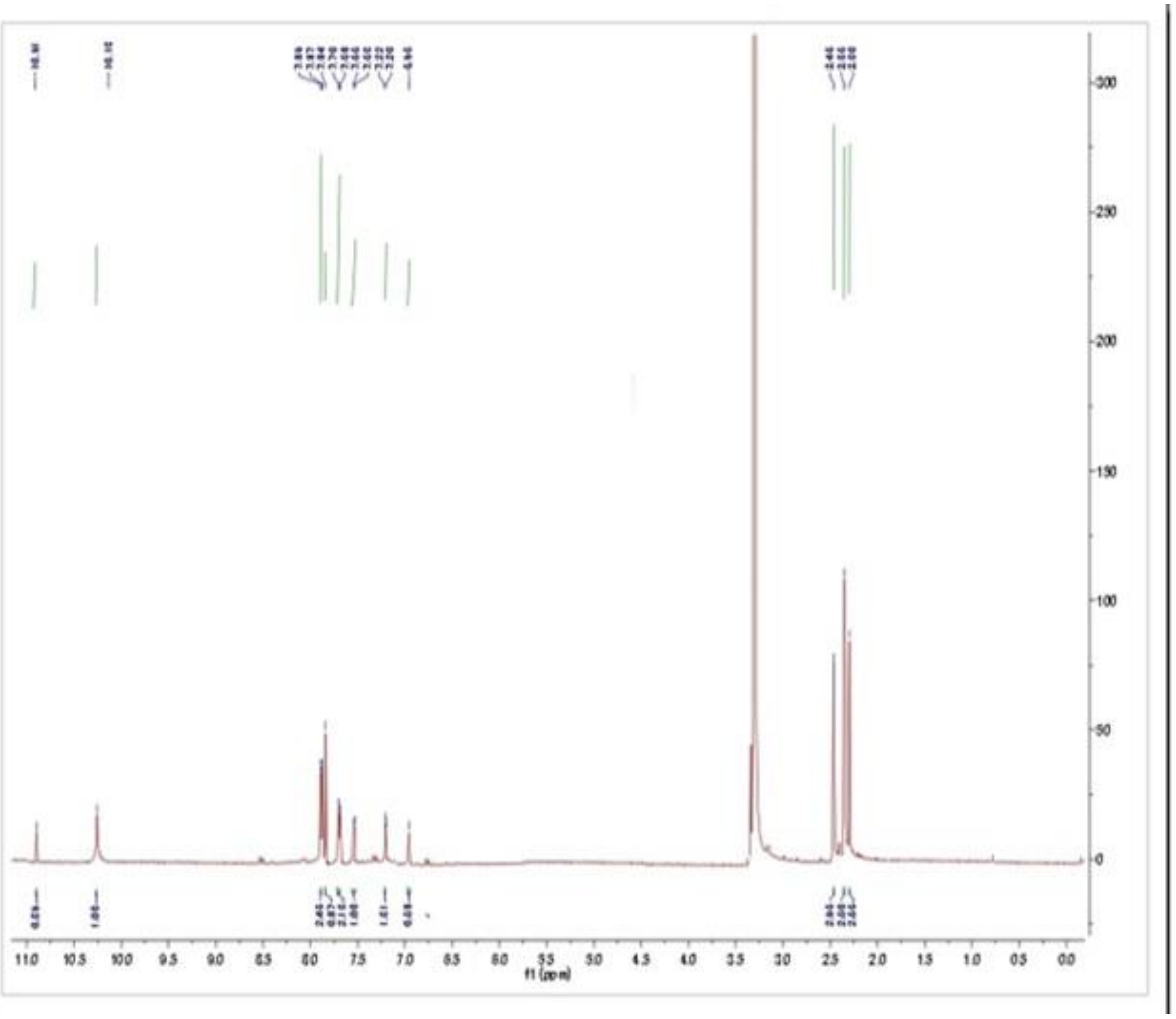

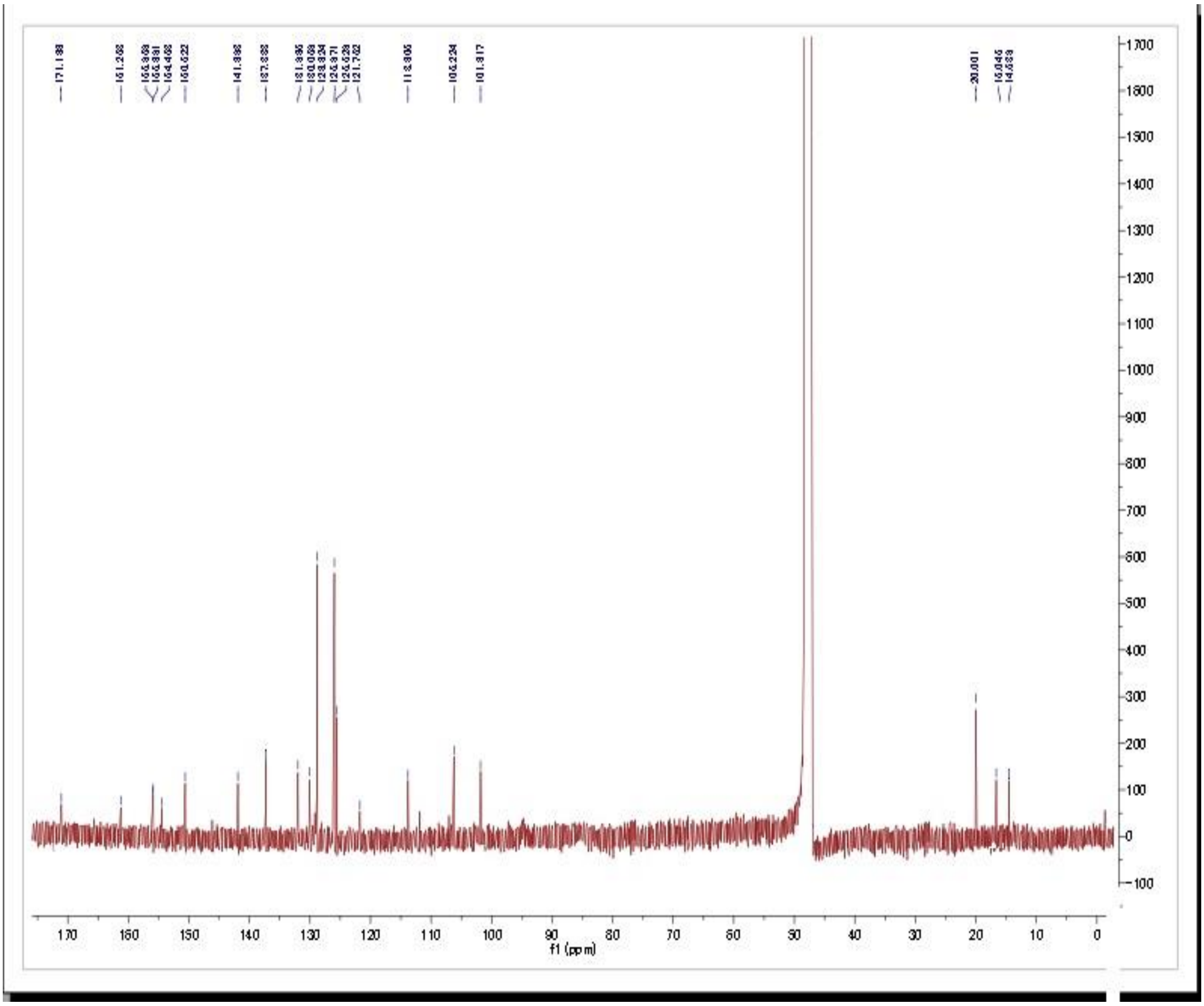

171.188
161.268
154.468
150.622
125.971
121.762
106.234
101.317
20.001
f1 (ppm)

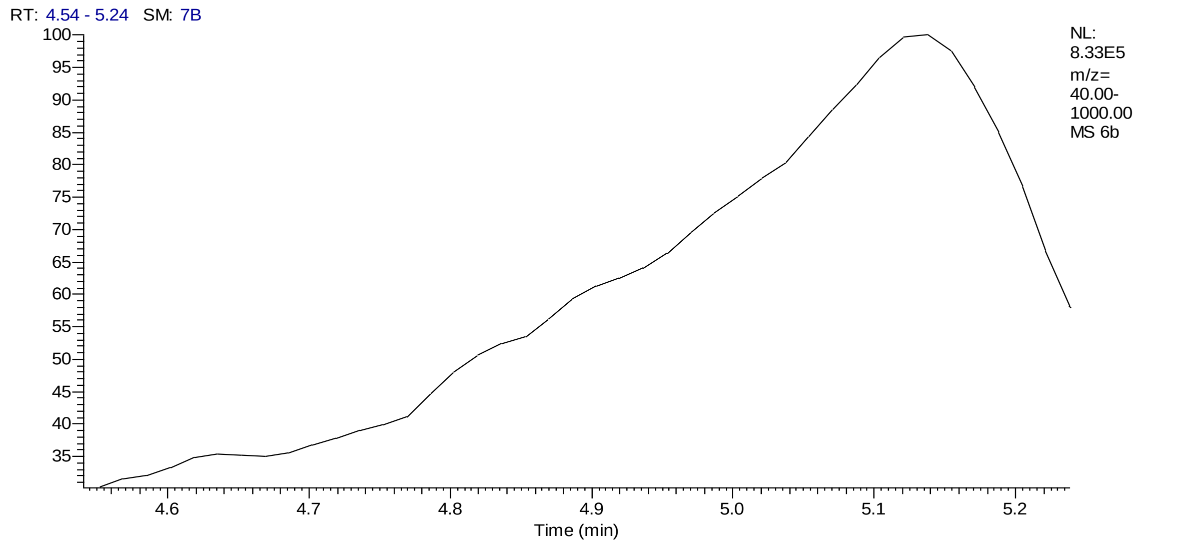
RT: 4.54 - 5.24 SM: 7B
NL:
8.33E5
m/z=
40.00-
1000.00
MS 6b
Time (min)

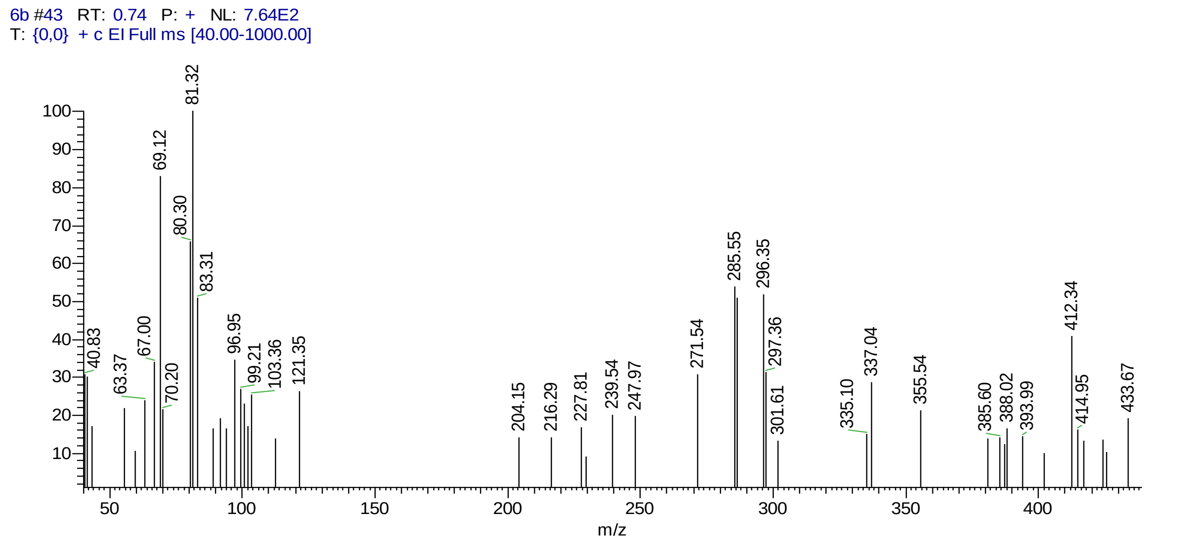
6b #43 RT: 0.74 P: + NL: 7.64E2
T: {0,0} + c EI Full ms [40.00-1000.00]
81.32
69.12
80.30
83.31
40.83
63.37
67.00
70.20
96.95
99.21
103.36
121.35
204.15
216.29
227.81
239.54
247.97
271.54
285.55
296.35
297.36
301.61
335.10
337.04
355.54
385.60
388.02
393.99
412.34
414.95
433.67
m/z

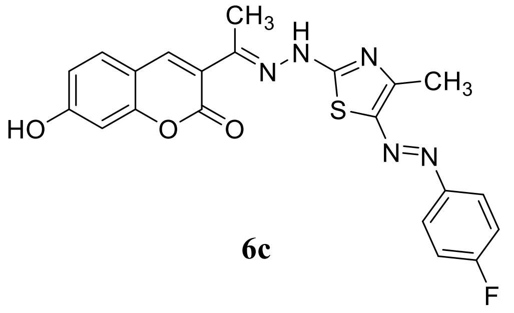

6c

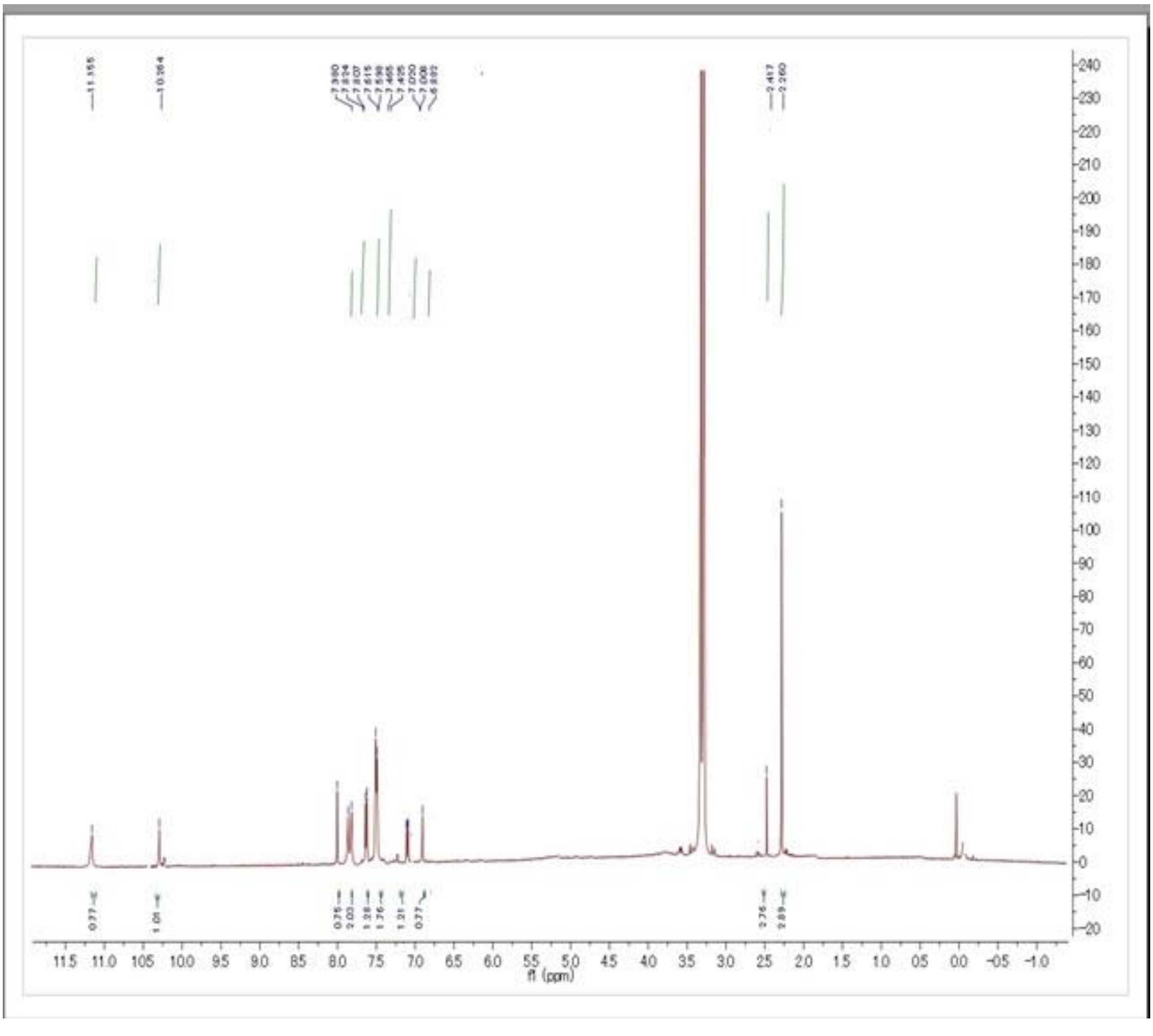

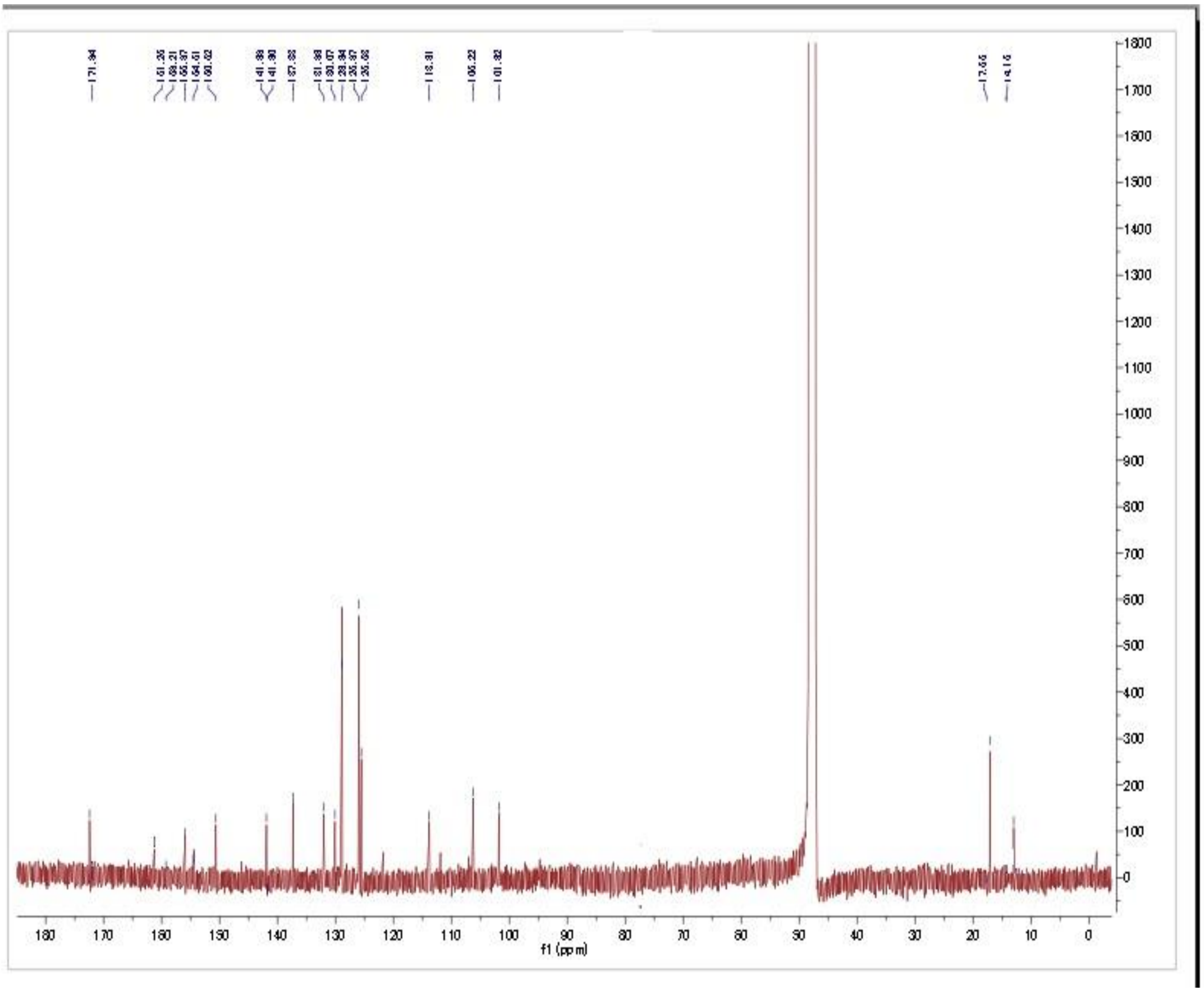

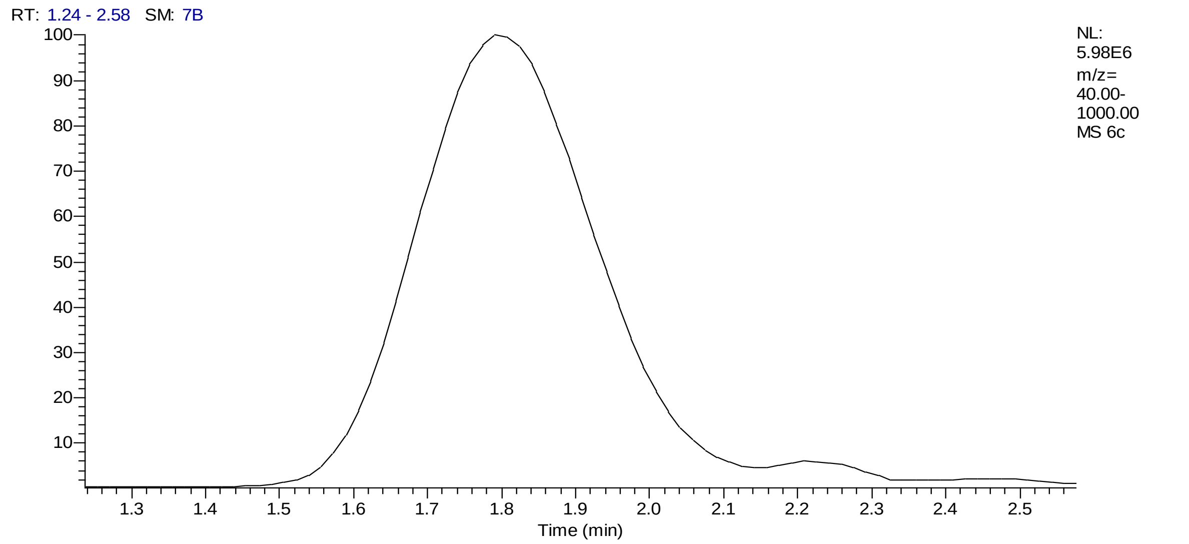
RT: 1.24 - 2.58 SM: 7B
NL: 5.98E6 m/z= 40.00-1000.00 MS 6c
100
90
80
70
60
50
40
30
20
10
1.3
1.4
1.5
1.6
1.7
1.8
1.9
2.0
2.1
2.2
2.3
2.4
2.5
Time (min)

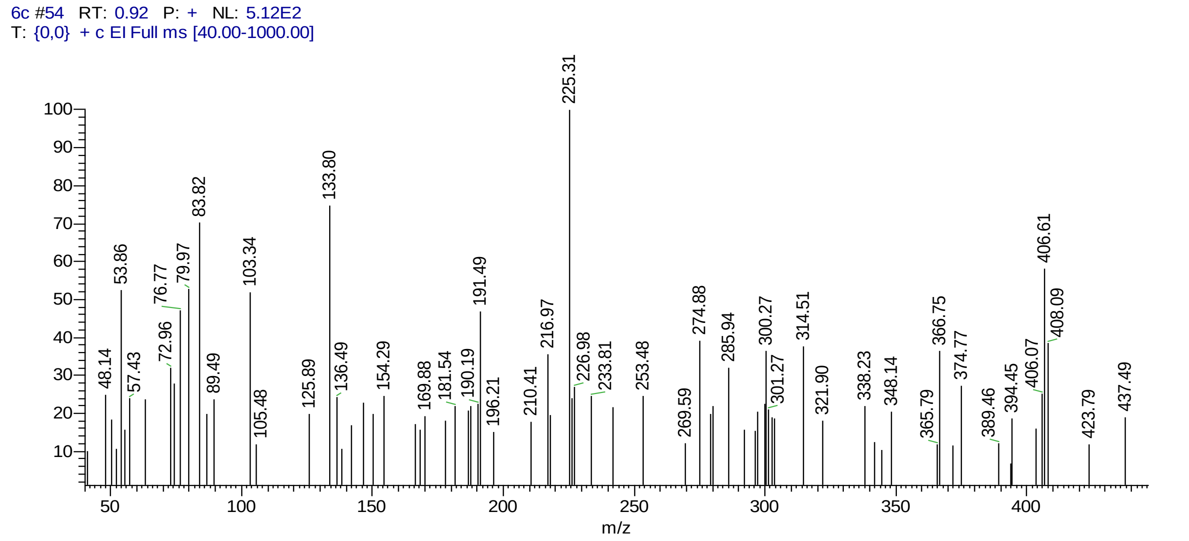
6c #54 RT: 0.92 P: + NL: 5.12E2
T: {0,0} + c EI Full ms [40.00-1000.00]
225.31
133.80
83.82
79.97
76.77
72.96
53.86
48.14
57.43
89.49
103.34
105.48
125.89
136.49
154.29
169.88
181.54
190.19
191.49
196.21
210.41
216.97
226.98
233.81
253.48
269.59
274.88
285.94
300.27
301.27
314.51
321.90
338.23
348.14
365.79
366.75
374.77
389.46
394.45
406.07
406.61
408.09
423.79
437.49
100
90
80
70
60
50
40
30
20
10
50
100
150
200
250
300
350
400
m/z

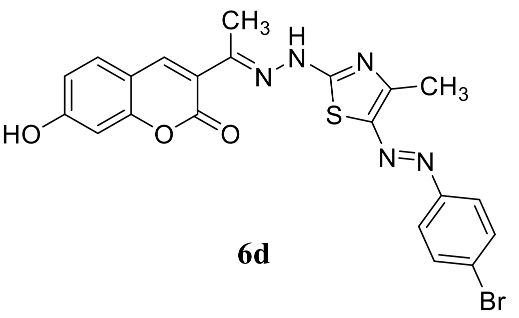

6d

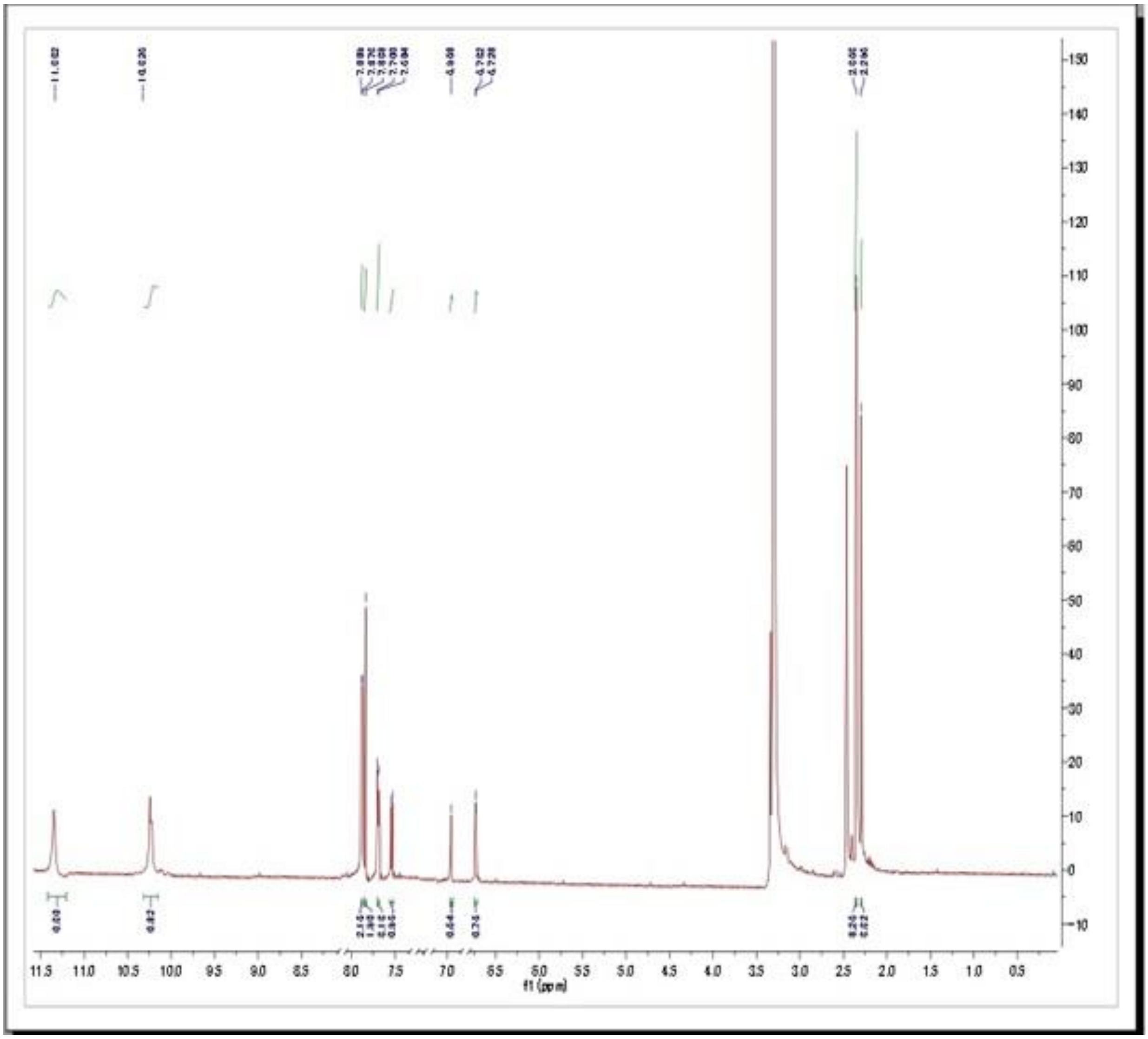

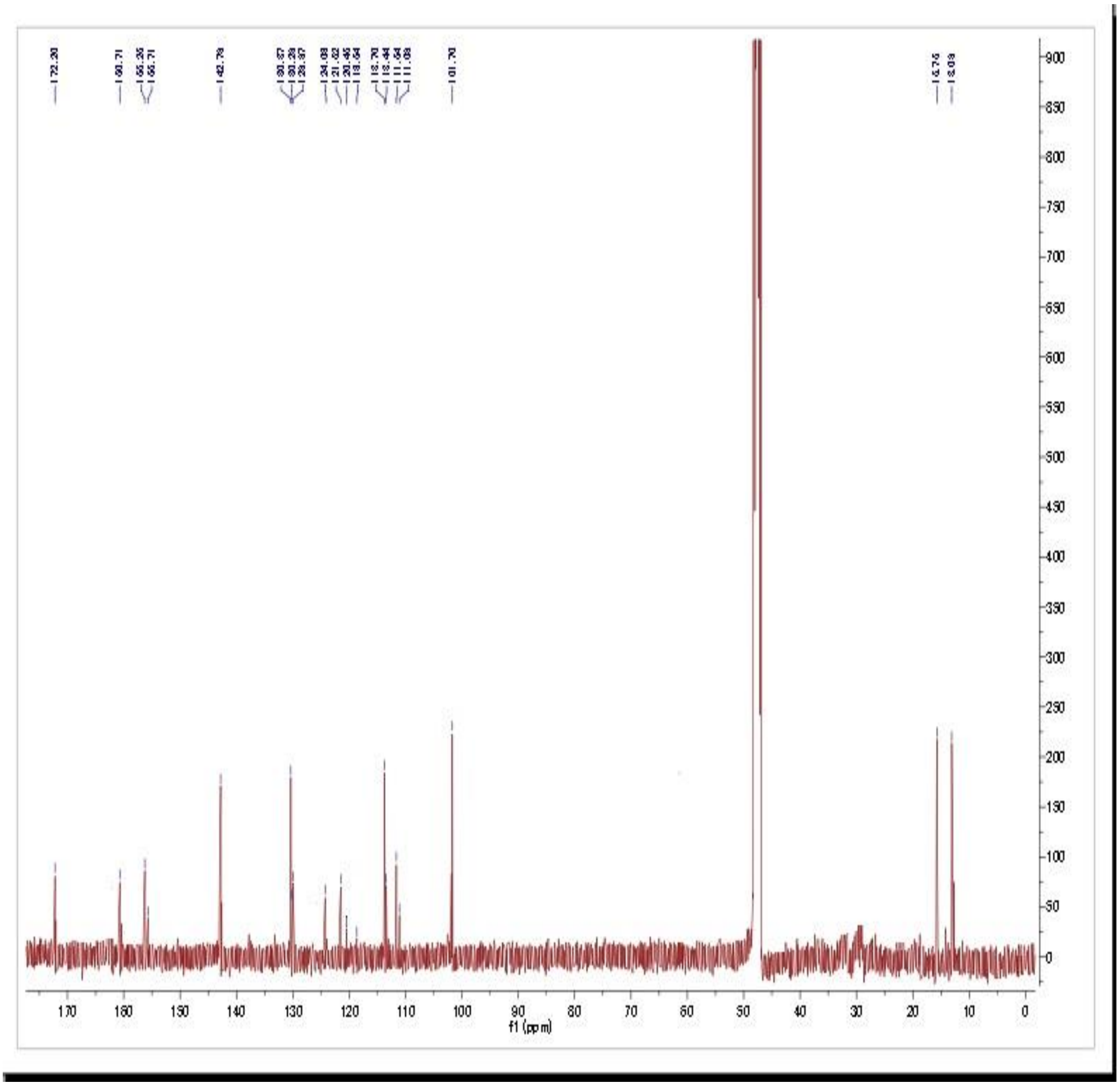

172.20
160.71
156.26
155.71
142.78
130.57
130.28
129.97
124.08
121.62
120.46
119.64
113.70
113.44
111.54
111.08
101.70
15.75
13.09
f1 (ppm)

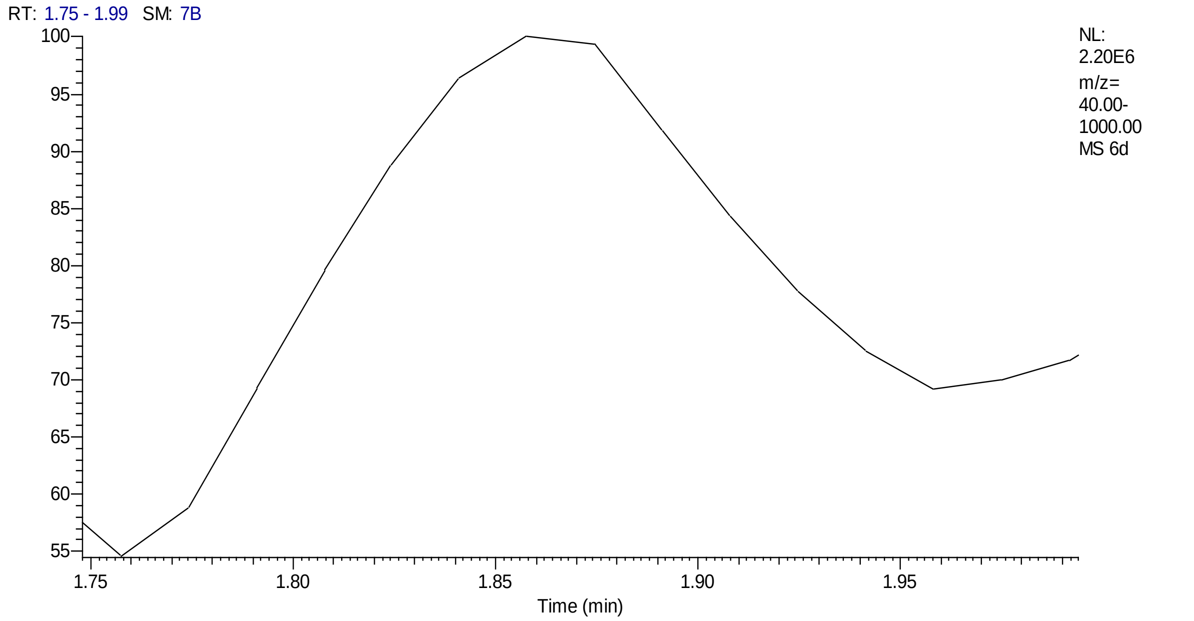
RT: 1.75 - 1.99 SM: 7B
NL:
2.20E6
m/z=
40.00-
1000.00
MS 6d
Time (min)

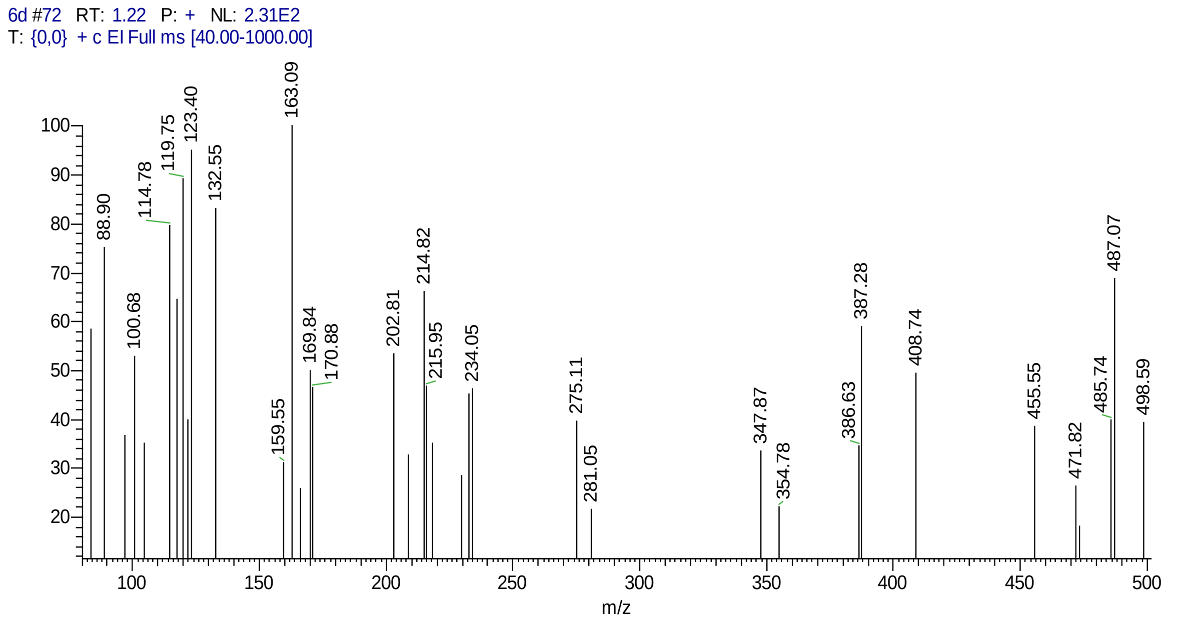
6d #72 RT: 1.22 P: + NL: 2.31E2
T: {0,0} + c EI Full ms [40.00-1000.00]
88.90
100.68
114.78
119.75
123.40
132.55
159.55
163.09
169.84
170.88
202.81
214.82
215.95
234.05
275.11
281.05
347.87
354.78
386.63
387.28
408.74
455.55
471.82
485.74
487.07
498.59
m/z

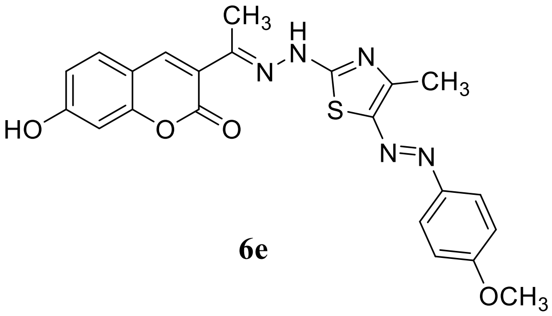

6e

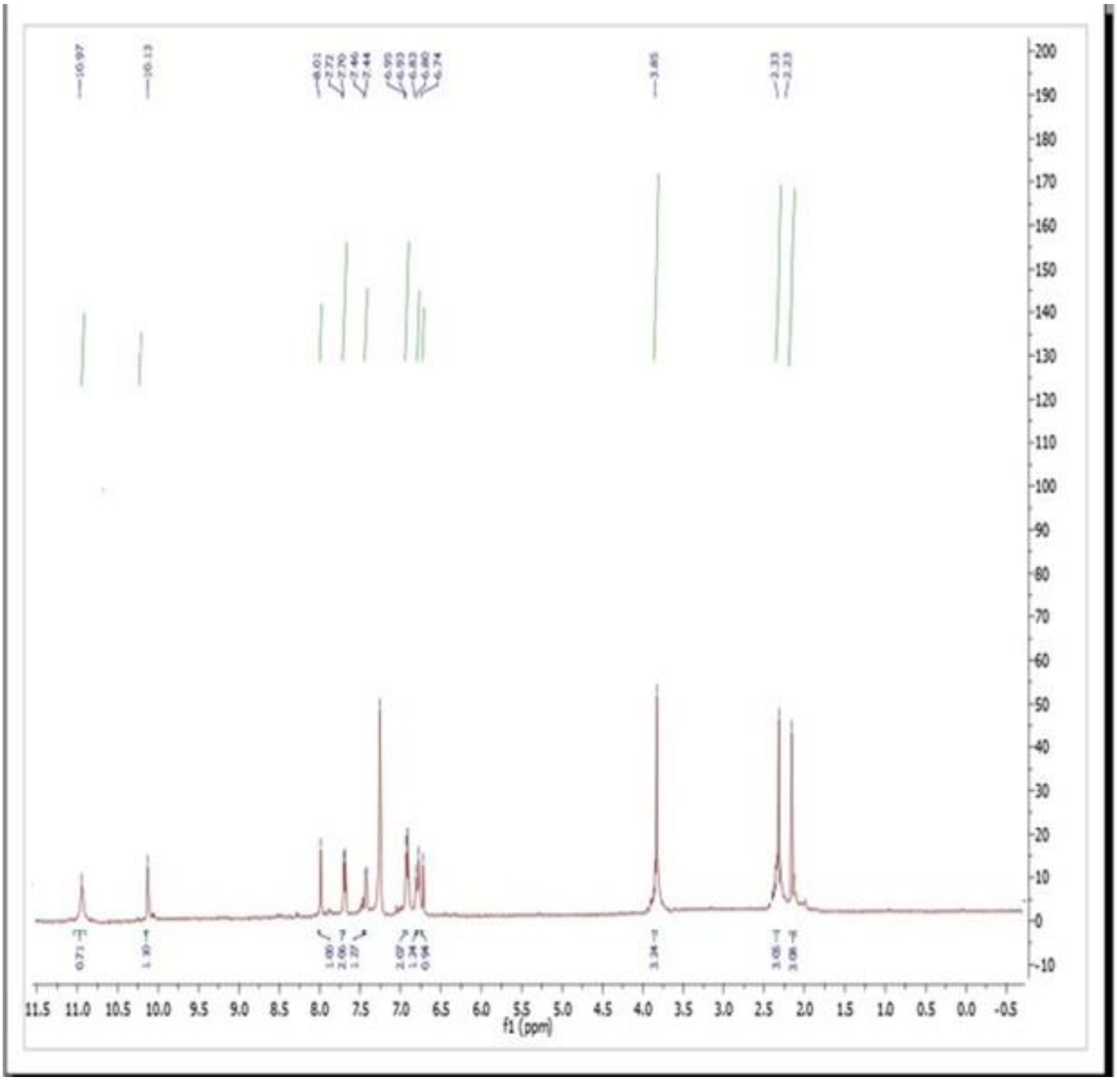

10.97
10.13
8.01
7.72
7.70
7.46
7.44
6.95
6.93
6.83
6.80
6.74
3.85
2.33
2.23
f1 (ppm)

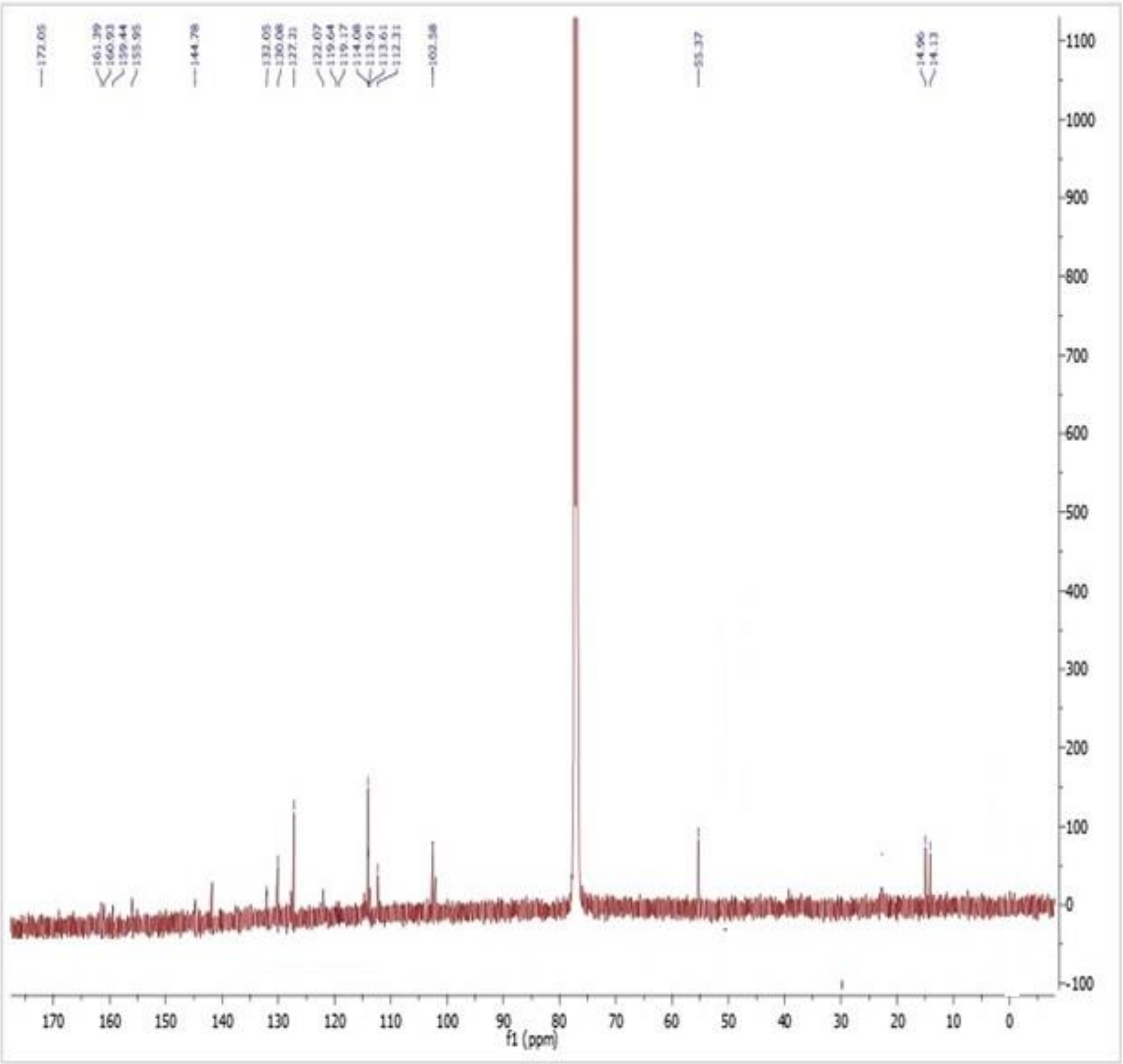
172.05
161.39
160.93
159.44
155.95
144.78
132.05
130.06
127.31
122.07
119.64
119.17
114.08
113.91
113.61
112.31
102.58
55.37
14.96
14.13
f1 (ppm)

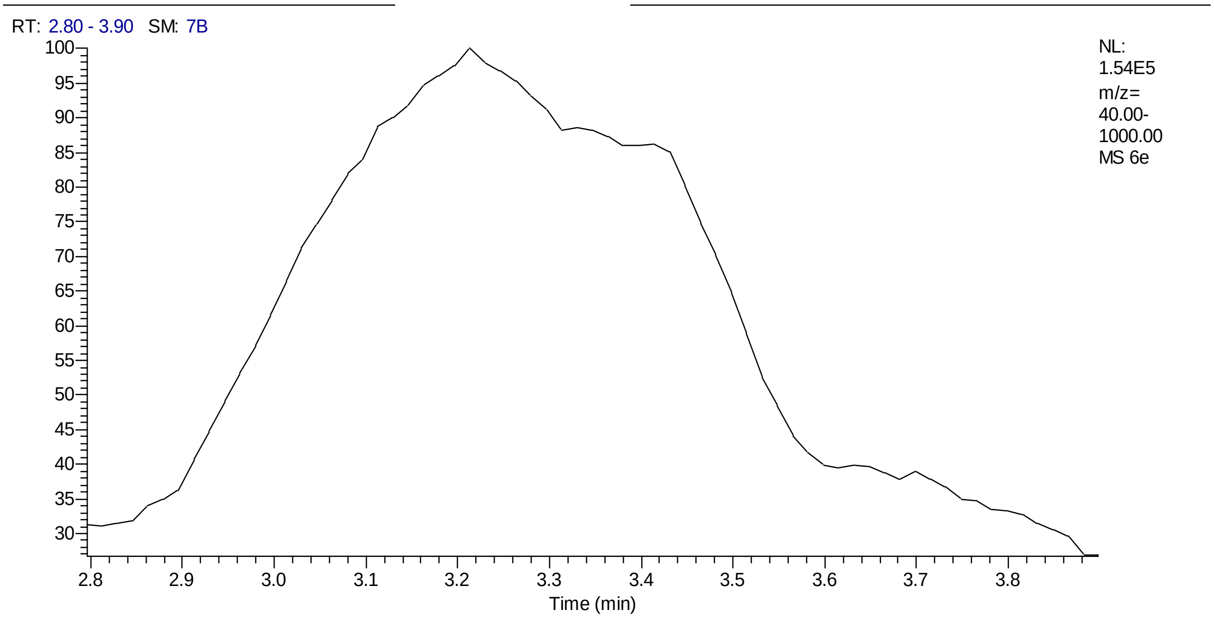
RT: 2.80 - 3.90 SM: 7B
NL: 1.54E5
m/z= 40.00-1000.00
MS 6e
Time (min)

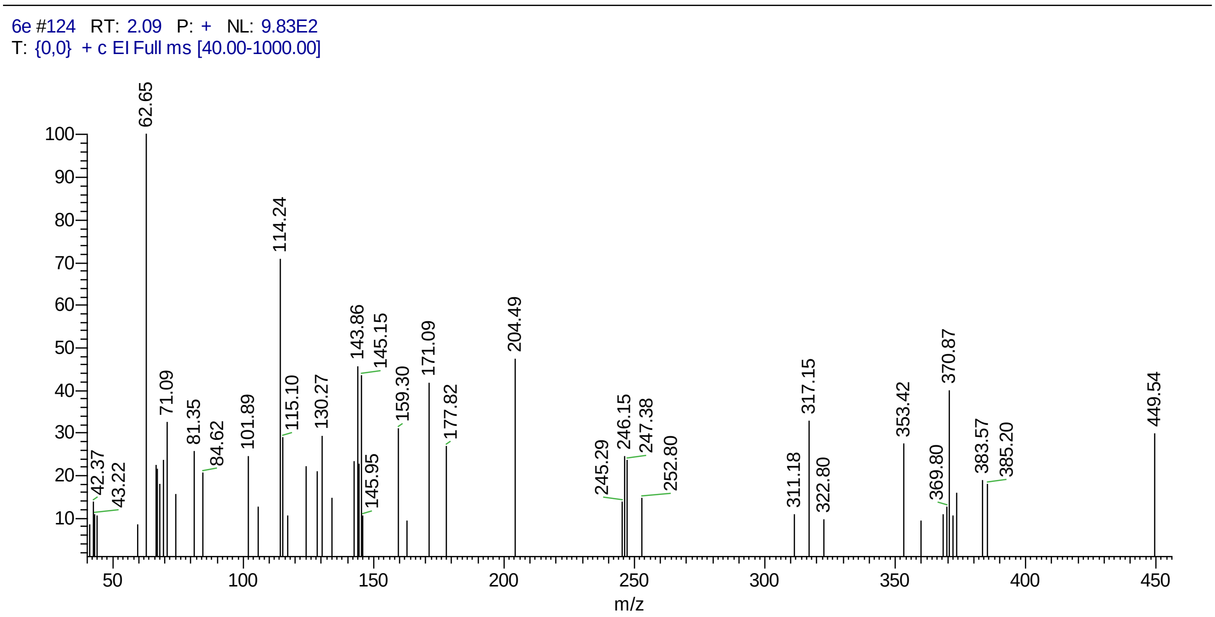
6e #124 RT: 2.09 P: + NL: 9.83E2
T: {0,0} + c EI Full ms [40.00-1000.00]
62.65
114.24
204.49
143.86
145.15
171.09
370.87
159.30
317.15
353.42
449.54
71.09
130.27
177.82
81.35
101.89
115.10
84.62
246.15
247.38
383.57
385.20
252.80
245.29
42.37
43.22
145.95
311.18
322.80
369.80
m/z

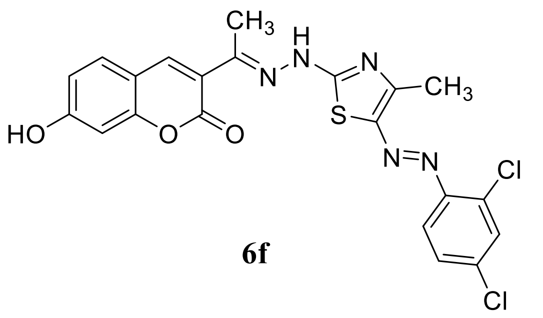

6f

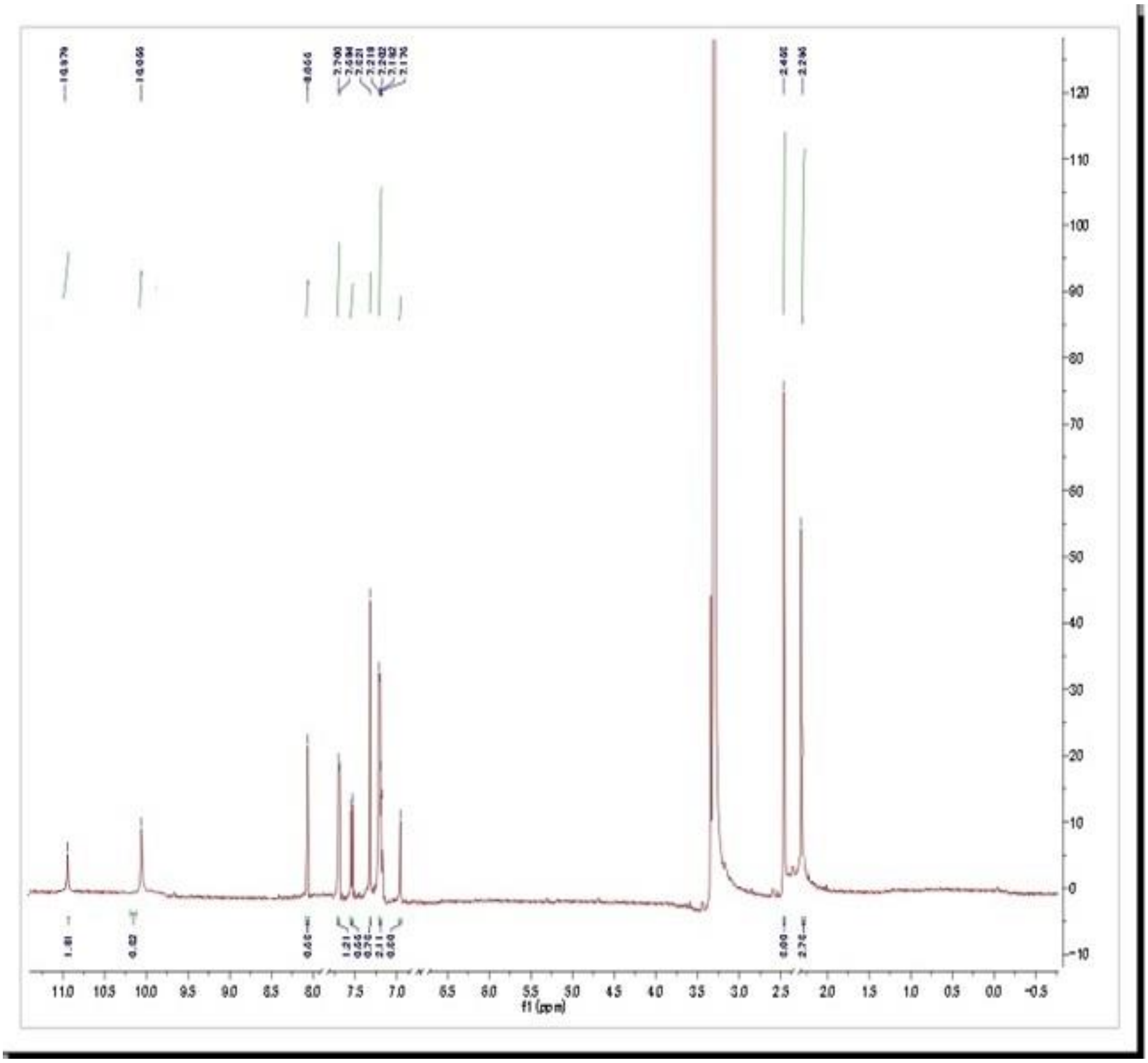

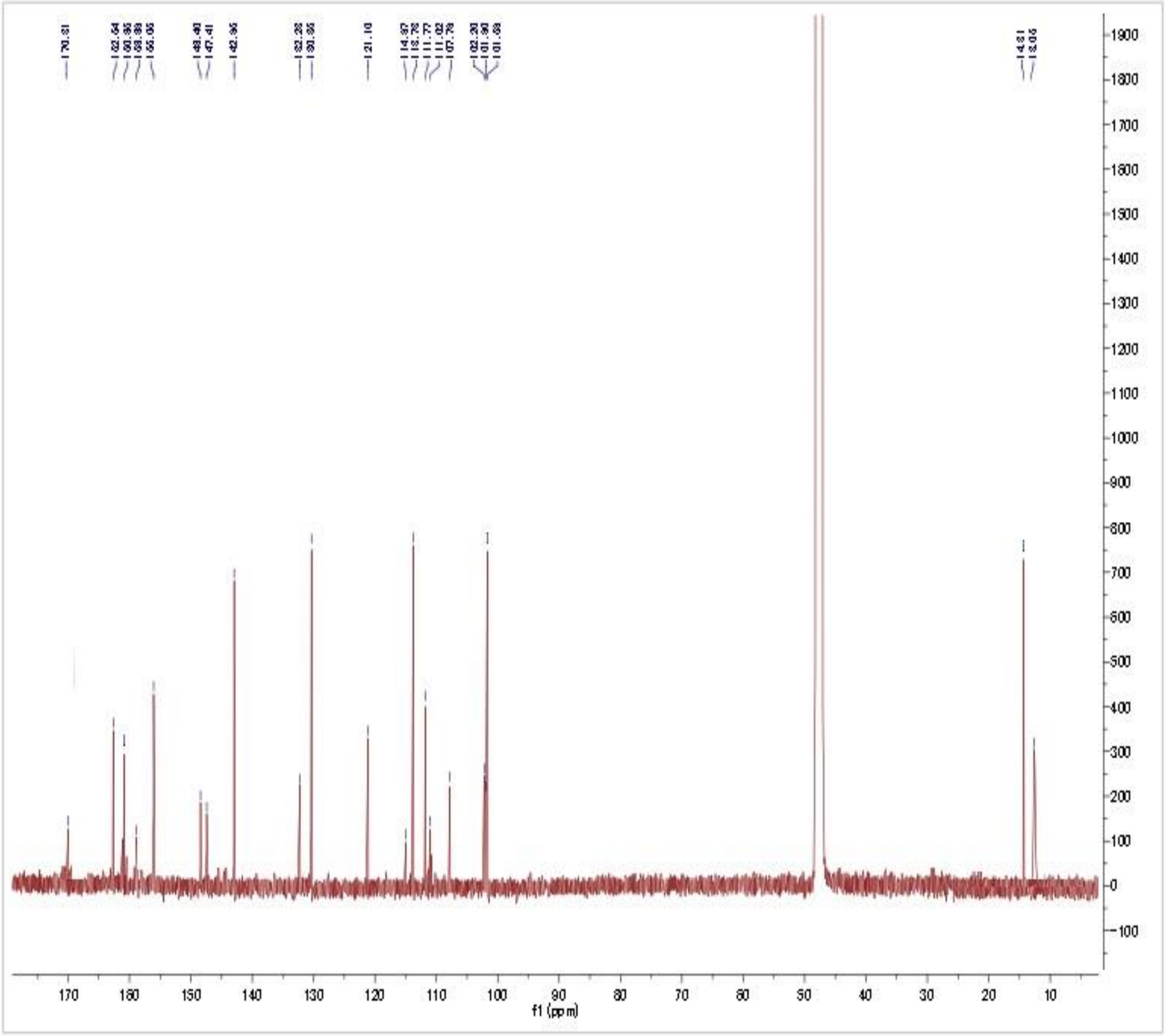
f1 (ppm)

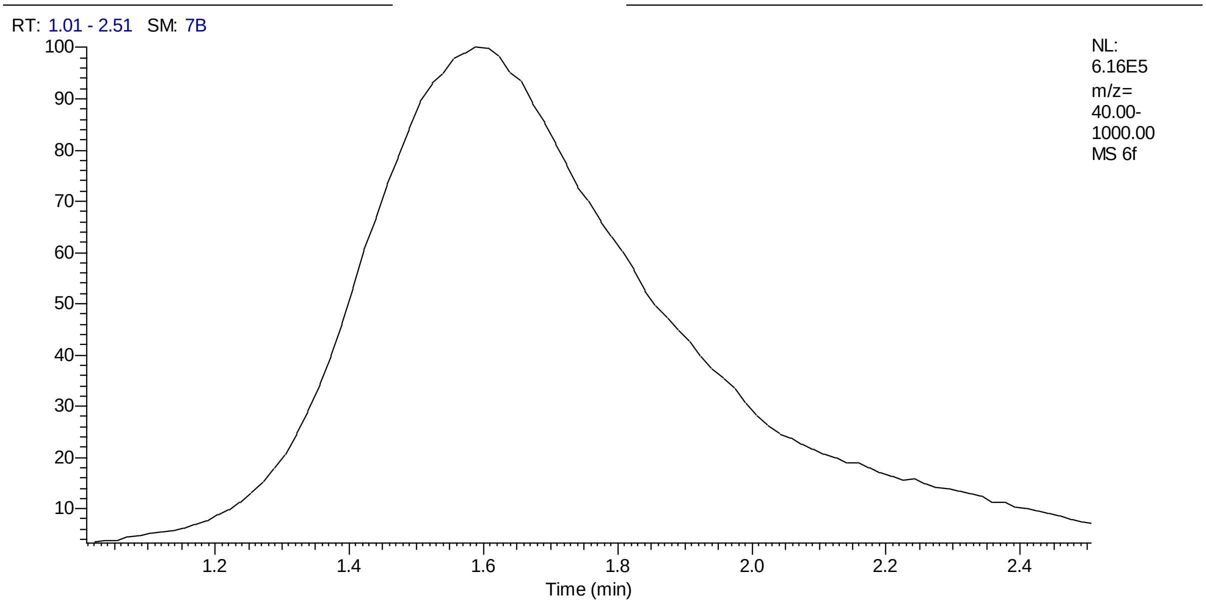
RT: 1.01 - 2.51 SM: 7B
NL: 6.16E5
m/z= 40.00-1000.00
MS 6f
Time (min)

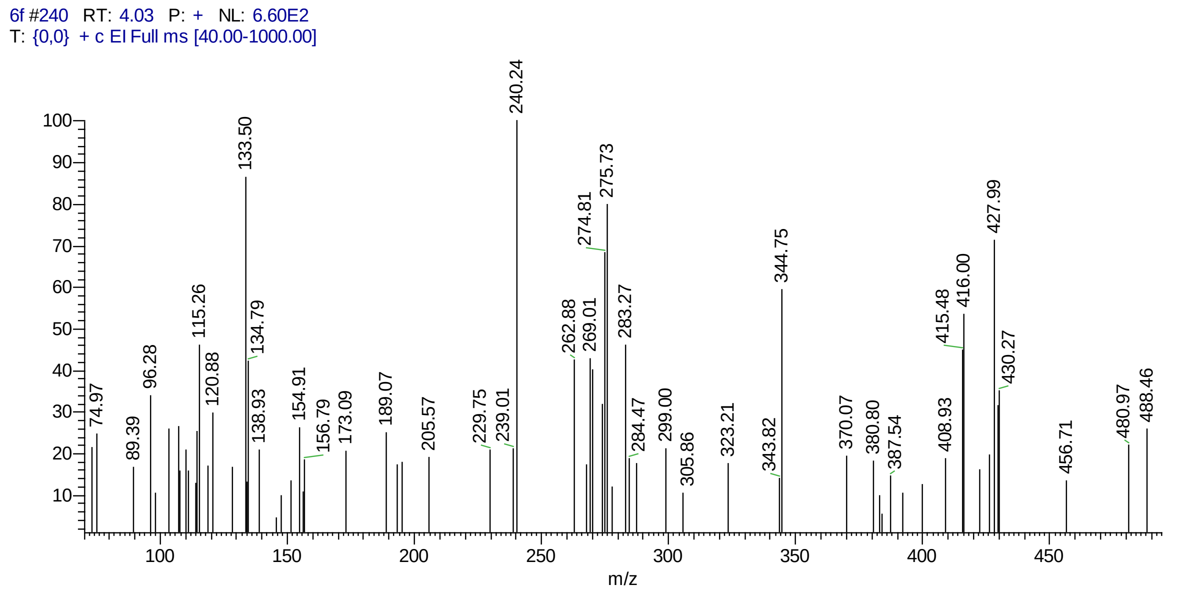
6f #240 RT: 4.03 P: + NL: 6.60E2
T: {0,0} + c EI Full ms [40.00-1000.00]
240.24
133.50
275.73
274.81
427.99
344.75
416.00
415.48
115.26
134.79
283.27
262.88
269.01
430.27
96.28
120.88
154.91
189.07
488.46
480.97
74.97
89.39
138.93
156.79
173.09
205.57
229.75
239.01
284.47
299.00
305.86
323.21
343.82
370.07
380.80
387.54
408.93
456.71
m/z

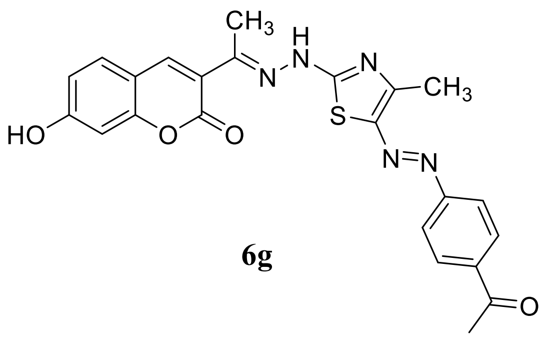

6g

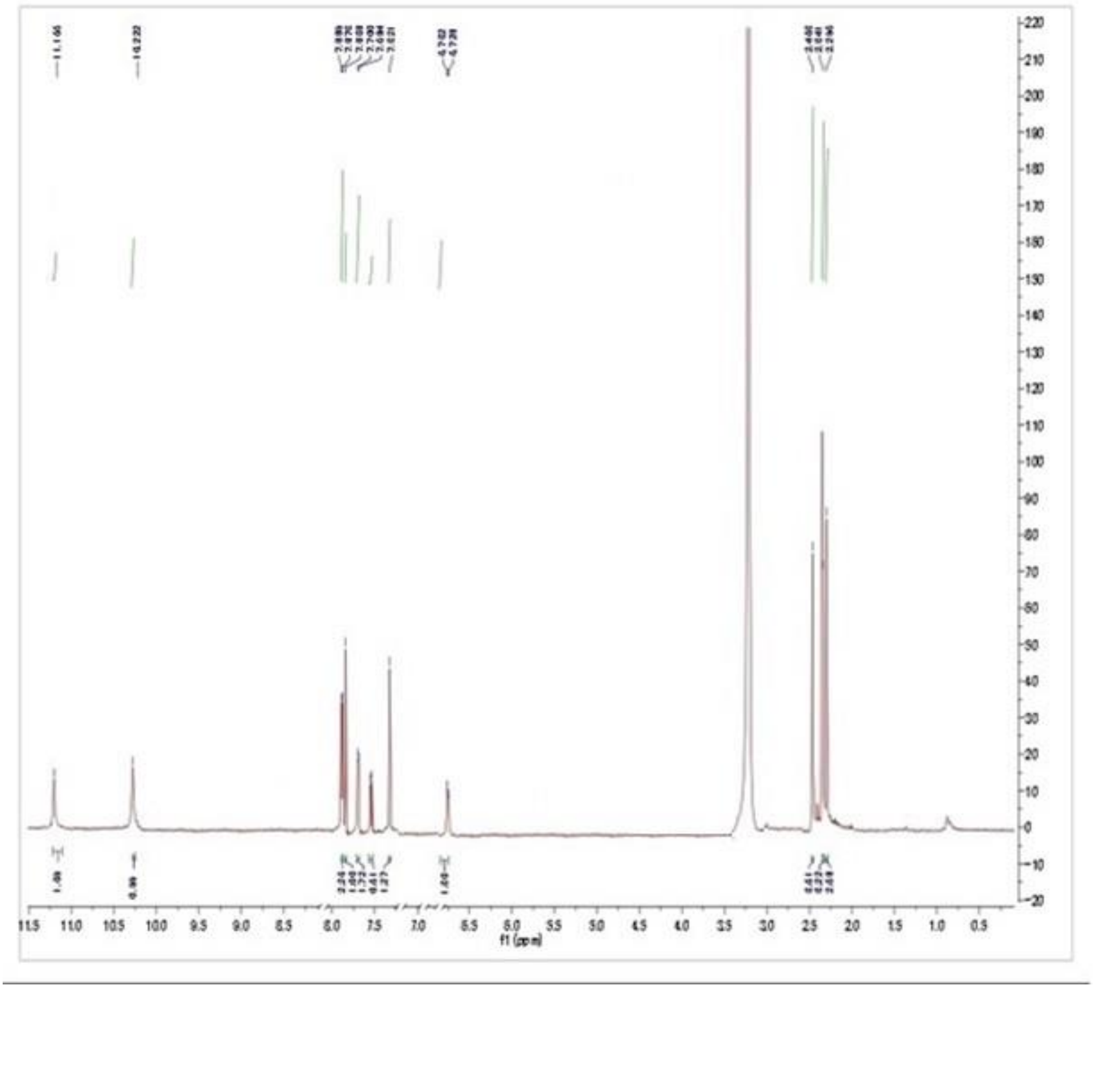

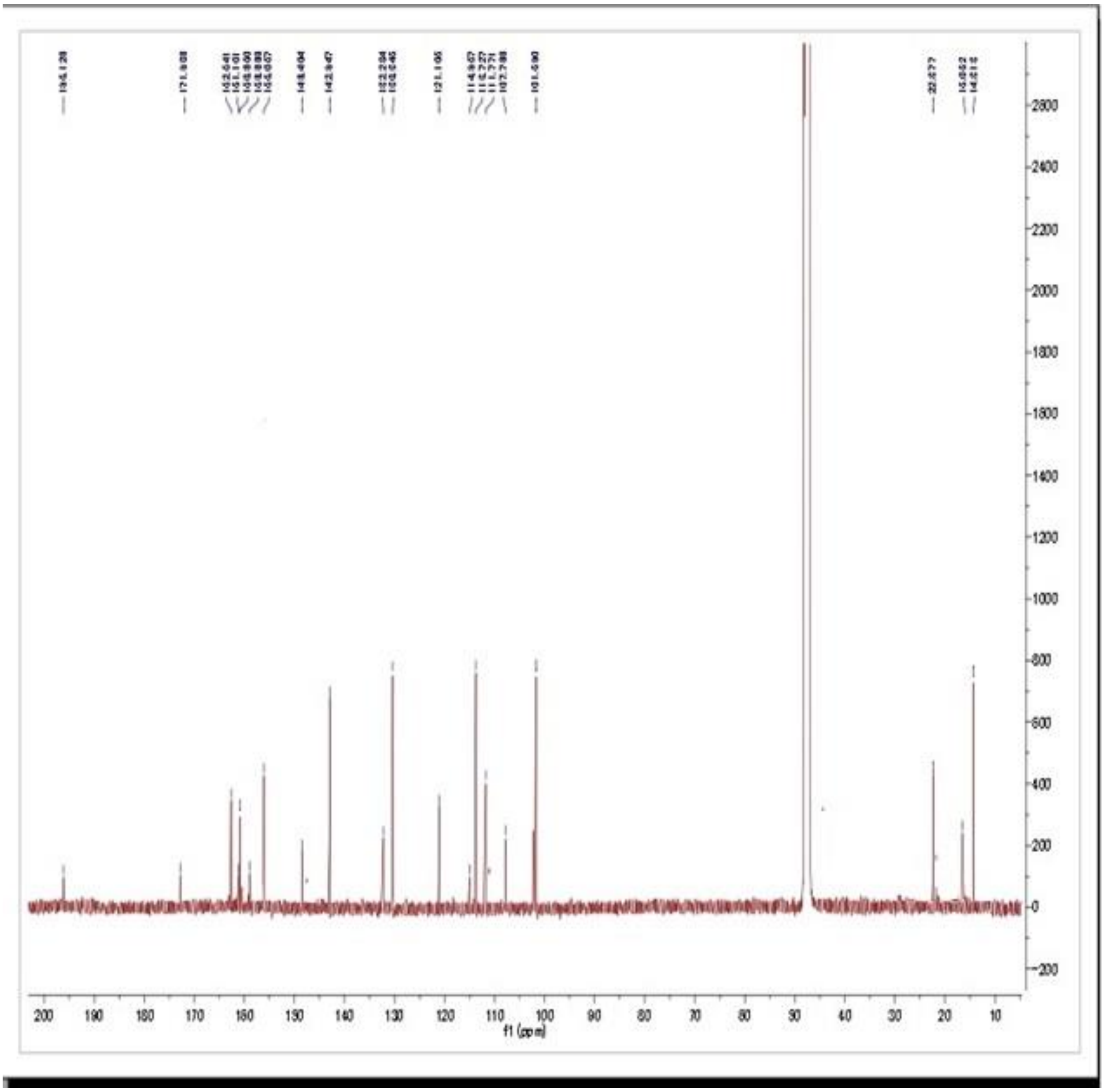
f1 (ppm)

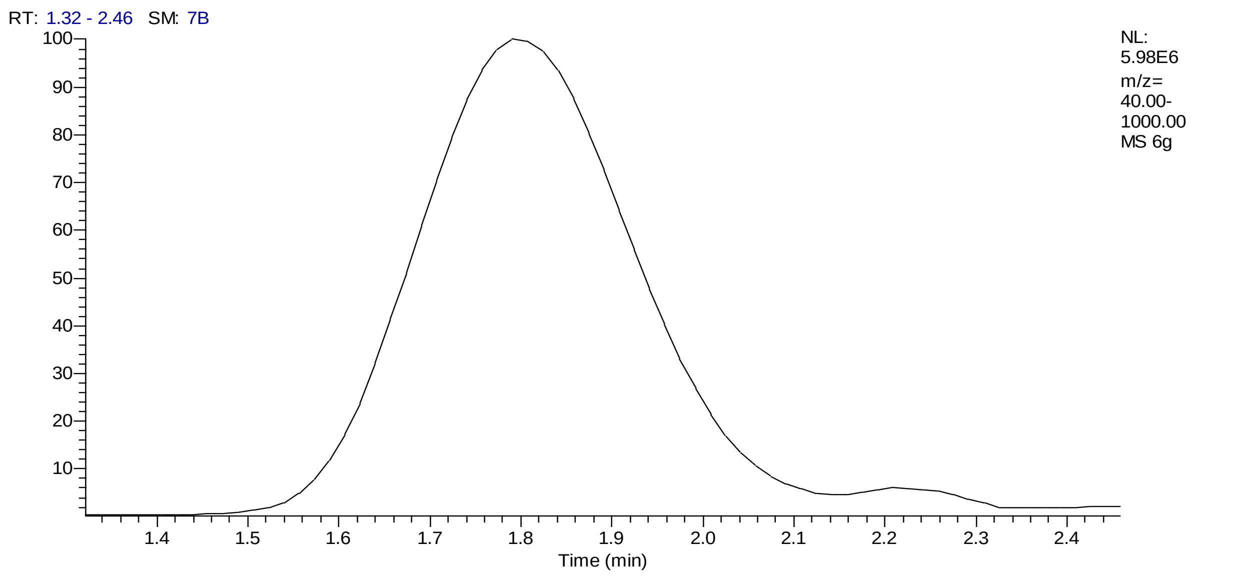
RT: 1.32 - 2.46 SM: 7B
NL: 5.98E6 m/z= 40.00-1000.00 MS 6g
Time (min)

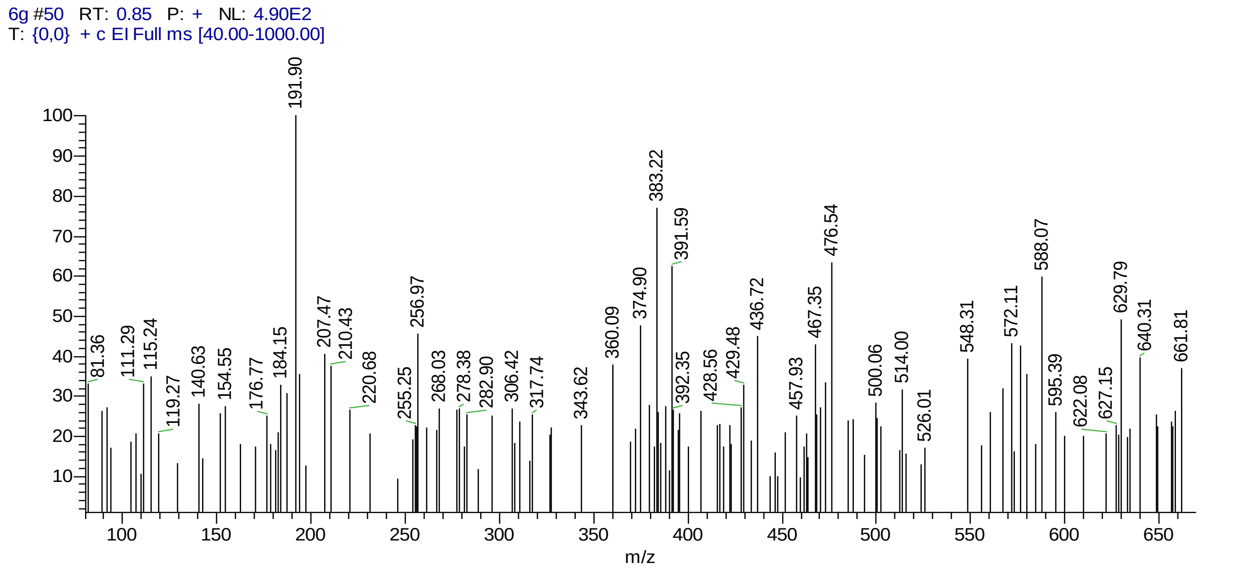
6g #50 RT: 0.85 P: + NL: 4.90E2
T: {0,0} + c EI Full ms [40.00-1000.00]
191.90
383.22
391.59
476.54
588.07
629.79
m/z

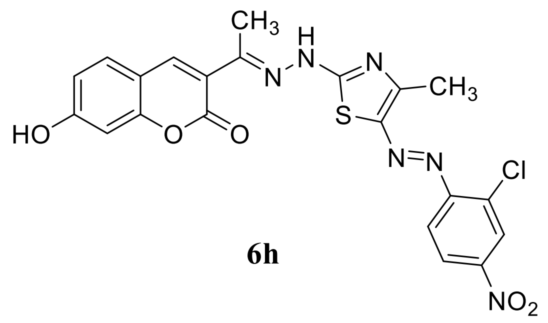

6h

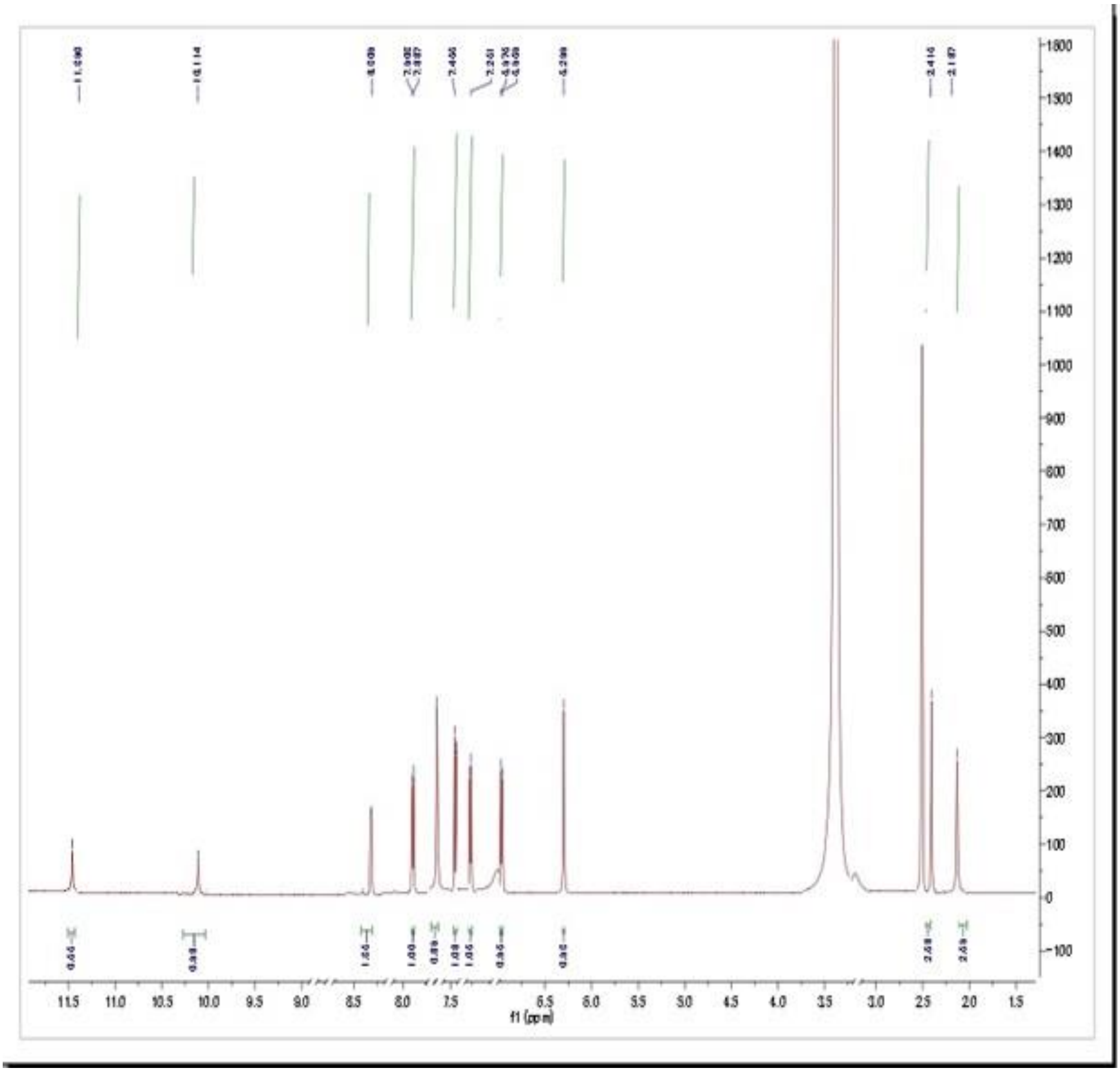

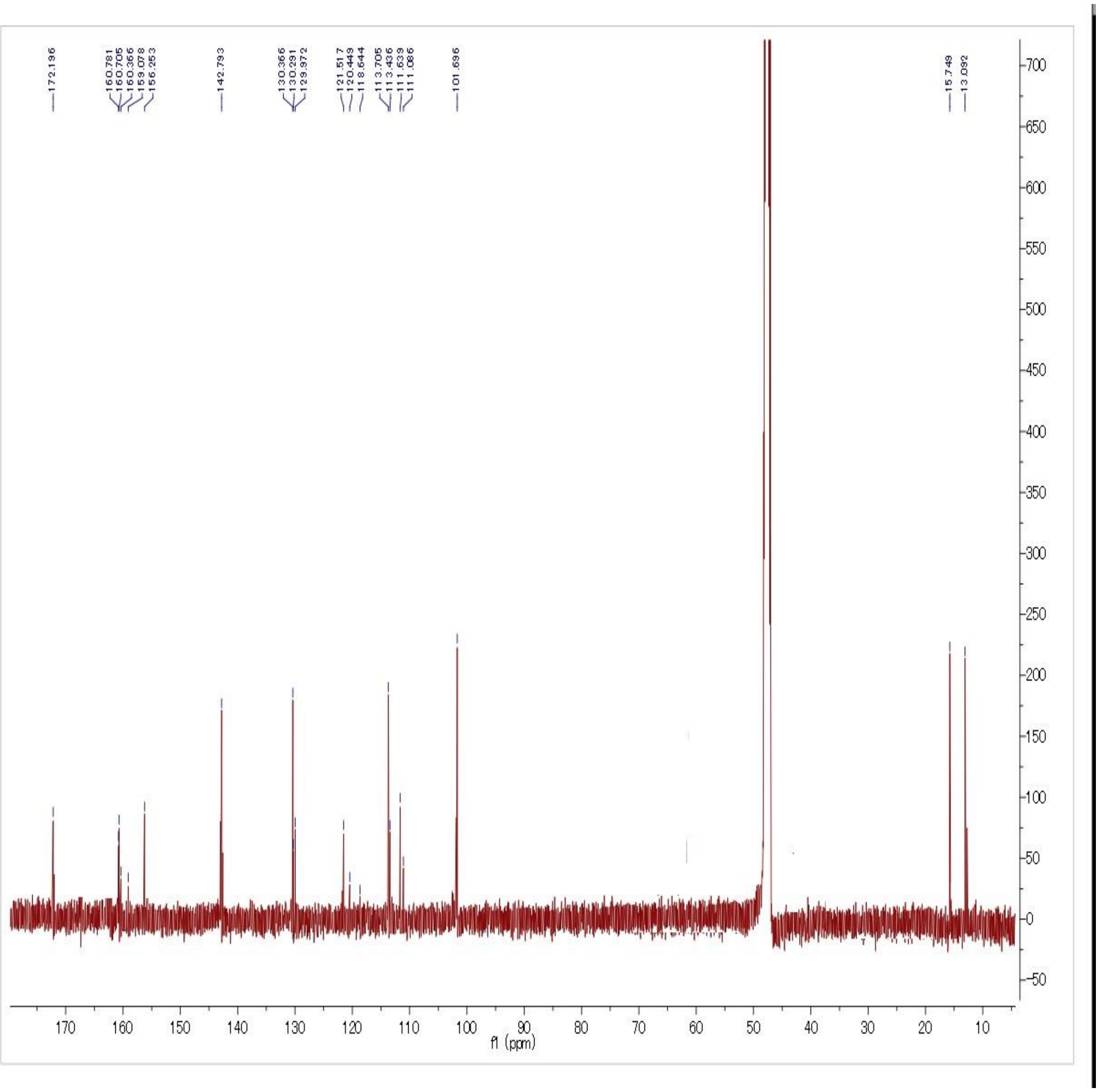

172.196
160.781
160.705
160.366
159.078
156.253
142.793
130.366
130.291
129.972
121.517
120.449
118.644
113.705
113.436
111.639
111.086
101.696
15.749
13.092
f1 (ppm)

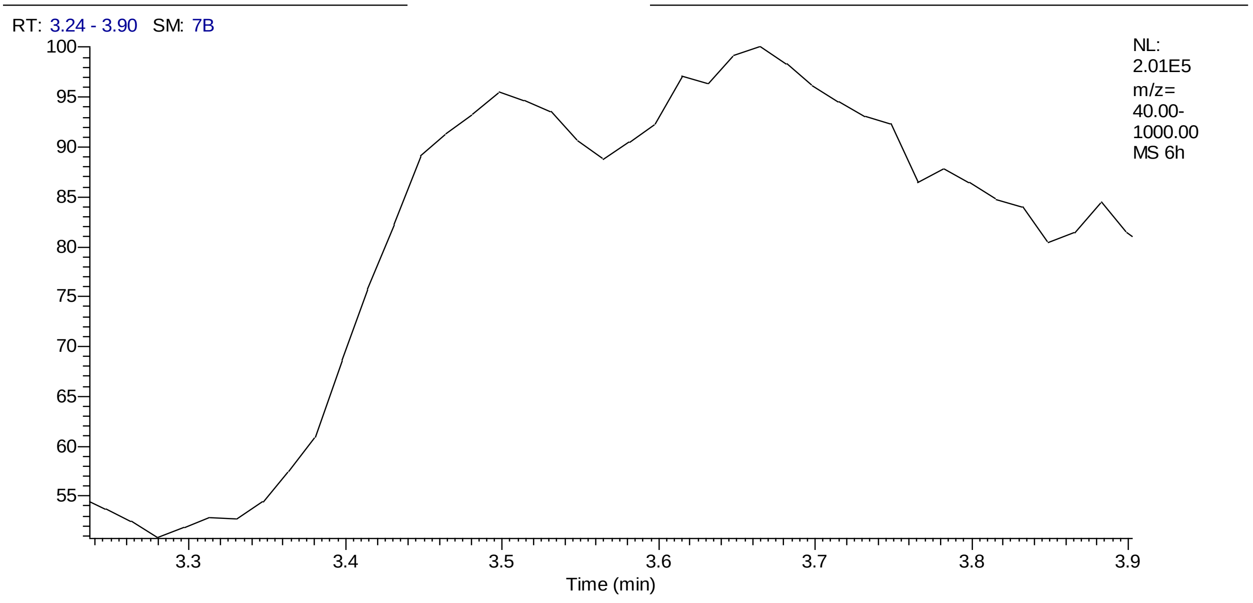
RT: 3.24 - 3.90 SM: 7B
NL: 2.01E5 m/z= 40.00-1000.00 MS 6h
Time (min)

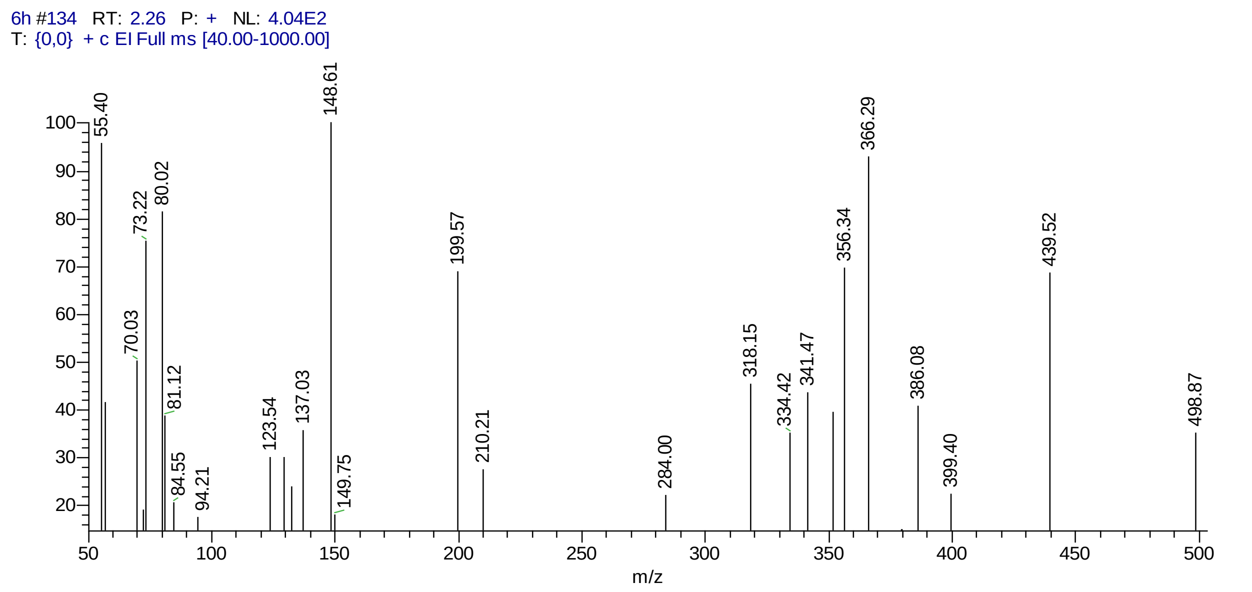
6h #134 RT: 2.26 P: + NL: 4.04E2
T: {0,0} + c EI Full ms [40.00-1000.00]
55.40
70.03
73.22
80.02
81.12
84.55
94.21
123.54
137.03
148.61
149.75
199.57
210.21
284.00
318.15
334.42
341.47
356.34
366.29
386.08
399.40
439.52
498.87
m/z

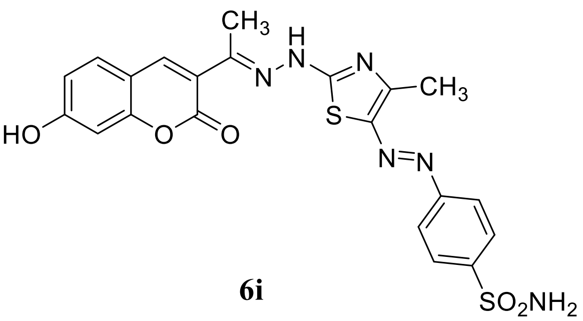
CH3
H
N
N
N
CH3
S
HO
O
O
N=N
SO2NH2

6i

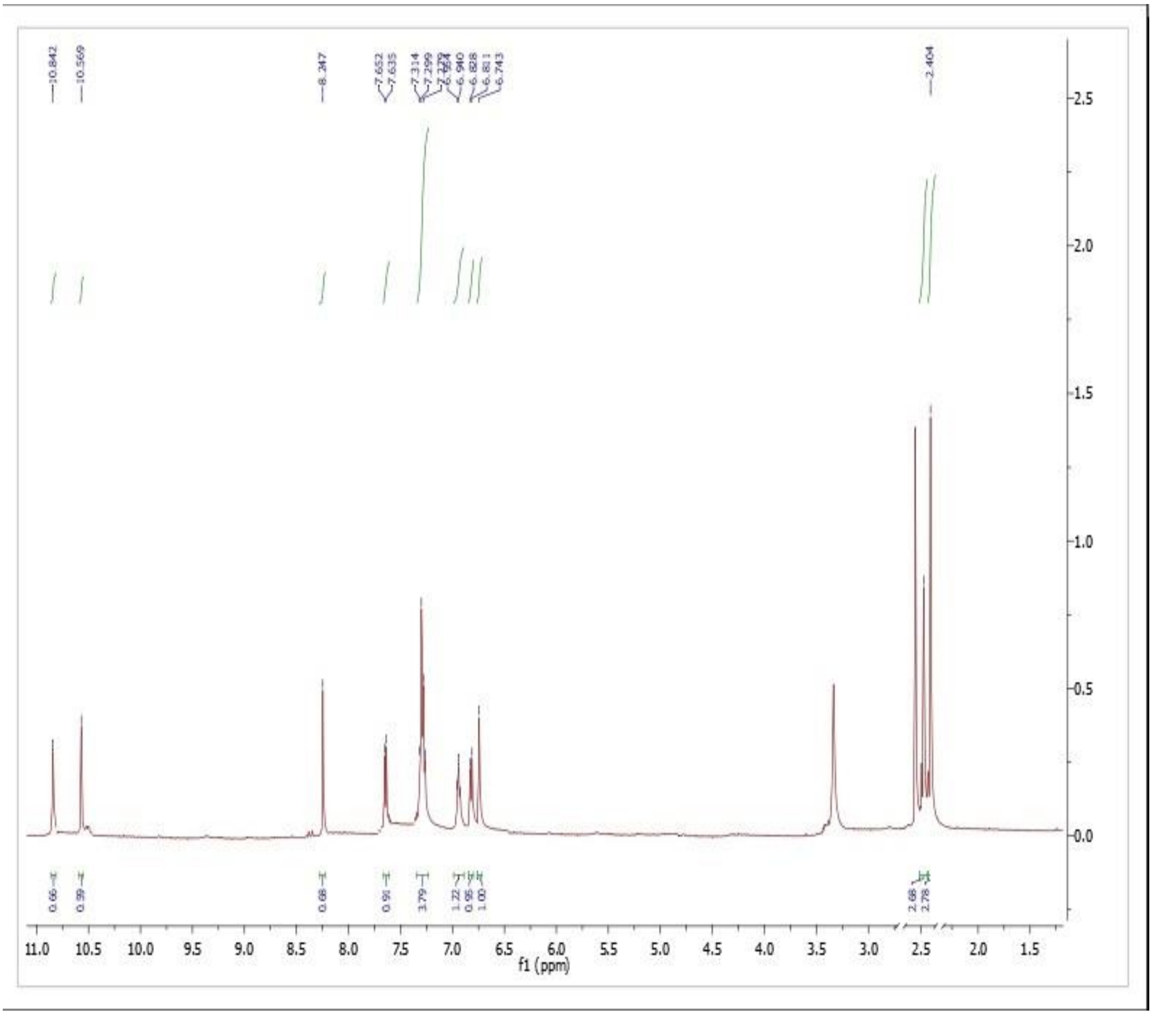
10.842
10.569
8.247
7.652
7.635
7.314
7.299
6.940
6.828
6.811
6.743
2.404
0.66
0.99
0.68
0.91
3.79
1.22
0.95
1.00
2.68
2.78
f1 (ppm)

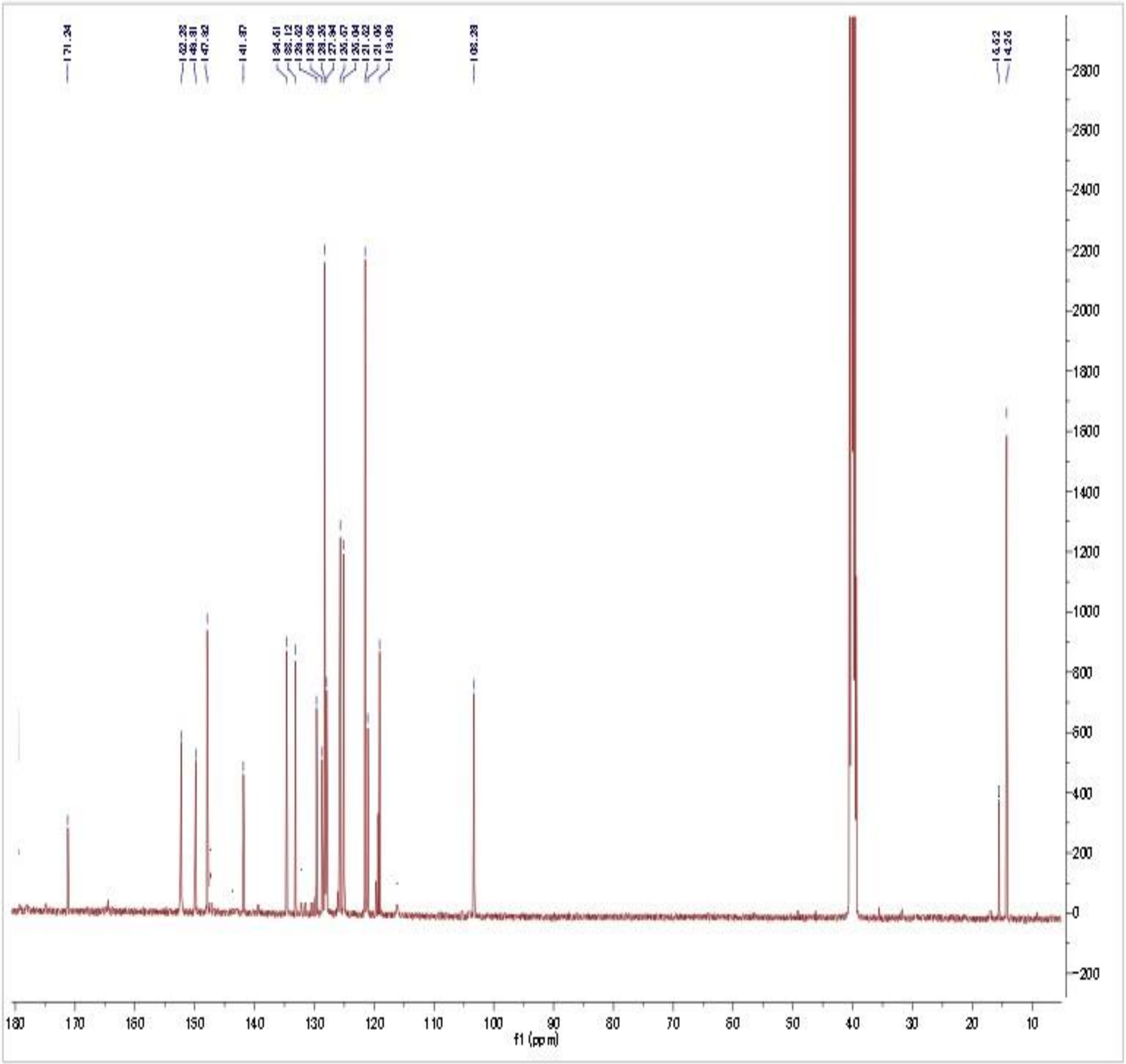
171.34
152.28
149.81
147.82
141.97
134.51
133.12
129.62
128.69
128.26
127.94
125.67
125.04
121.62
121.06
119.09
103.28
15.62
14.26
f1 (ppm)

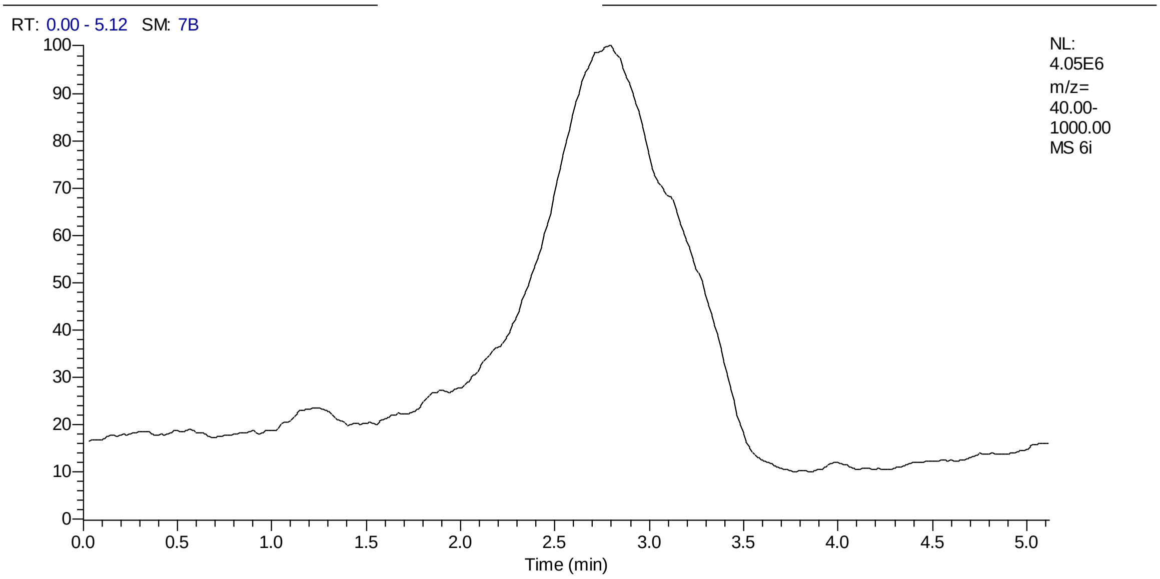

RT: 0.00 - 5.12 SM: 7B
NL: 4.05E6 m/z= 40.00-1000.00 MS 6i
Time (min)

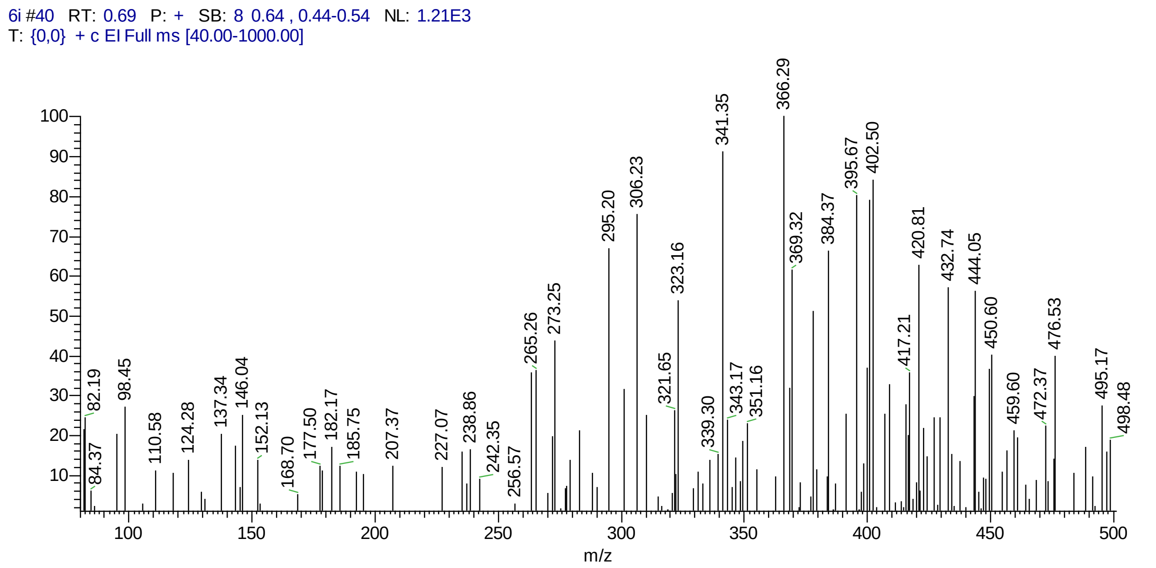

6i #40 RT: 0.69 P: + SB: 8 0.64 , 0.44-0.54 NL: 1.21E3
T: {0,0} + c EI Full ms [40.00-1000.00]
366.29
341.35
306.23
402.50
395.67
295.20
384.37
369.32
420.81
432.74
444.05
323.16
273.25
265.26
450.60
476.53
417.21
98.45
146.04
495.17
321.65
82.19
137.34
343.17
351.16
339.30
459.60
472.37
498.48
124.28
152.13
182.17
110.58
207.37
227.07
238.86
177.50
185.75
242.35
168.70
84.37
256.57
m/z

Figure S1. Antibacterial activity of the targeted molecules using agar-well diffusion

Serratia fonticola	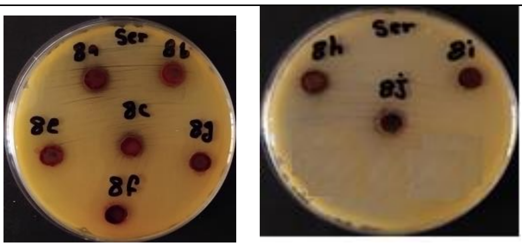 Group 8 (the target compounds 6a-i)
Campylobacter jejuni	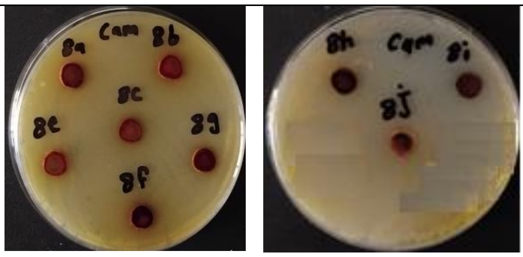 Group 8 (the target compounds 6a-i)
Enterococcus faecalis	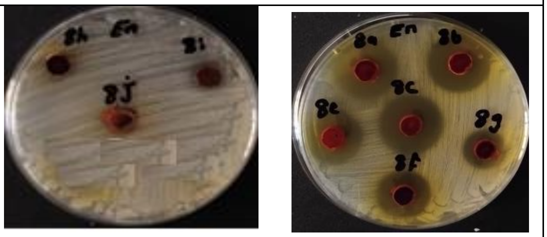 Group 8 (the target compounds 6a-i)
Achromobacter xylosoxidans	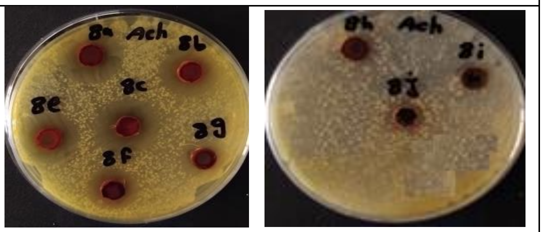 Group 8 (the target compounds 6a-i)

Figure S2:

Enterococcus faecalis	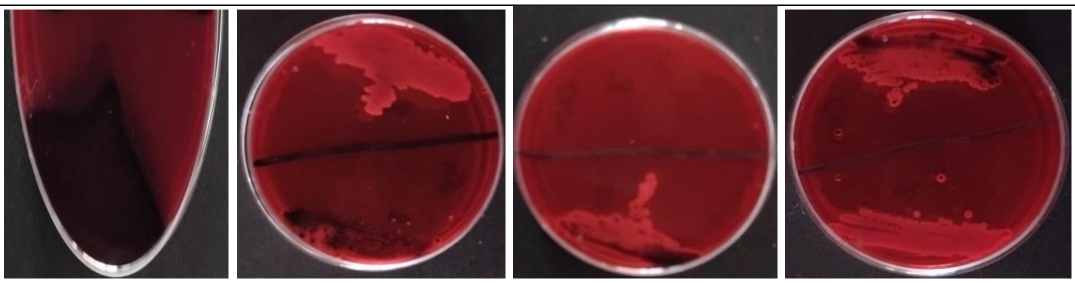
Achromobacter xylosoxidans	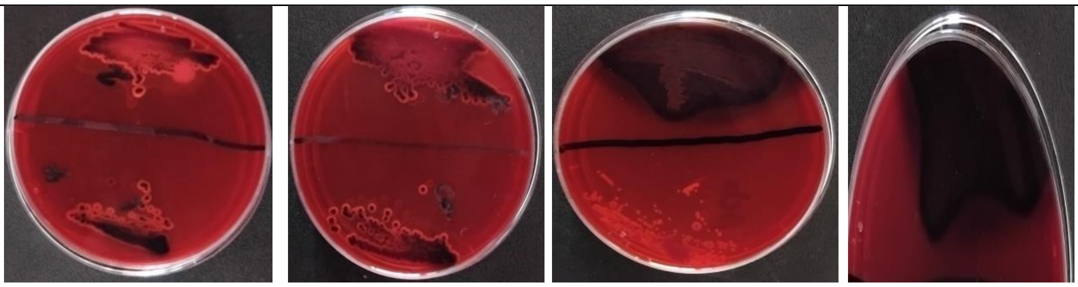
